# Delayed treatment effects, treatment switching and heterogeneous patient populations: How to design and analyze RCTs in oncology

**DOI:** 10.1002/pst.2062

**Published:** 2020-08-23

**Authors:** Robin Ristl, Nicolás M Ballarini, Heiko Götte, Armin Schüler, Martin Posch, Franz König

**Affiliations:** ^1^ Center for Medical Statistics, Informatics, and Intelligent Systems Medical University of Vienna Vienna Austria; ^2^ Merck Healthcare KGaA Darmstadt Germany

## Abstract

In the analysis of survival times, the logrank test and the Cox model have been established as key tools, which do not require specific distributional assumptions. Under the assumption of proportional hazards, they are efficient and their results can be interpreted unambiguously. However, delayed treatment effects, disease progression, treatment switchers or the presence of subgroups with differential treatment effects may challenge the assumption of proportional hazards. In practice, weighted logrank tests emphasizing either early, intermediate or late event times via an appropriate weighting function may be used to accommodate for an expected pattern of non‐proportionality. We model these sources of non‐proportional hazards via a mixture of survival functions with piecewise constant hazard. The model is then applied to study the power of unweighted and weighted log‐rank tests, as well as maximum tests allowing different time dependent weights. Simulation results suggest a robust performance of maximum tests across different scenarios, with little loss in power compared to the most powerful among the considered weighting schemes and huge power gain compared to unfavorable weights. The actual sources of non‐proportional hazards are not obvious from resulting populationwise survival functions, highlighting the importance of detailed simulations in the planning phase of a trial when assuming non‐proportional hazards.We provide the required tools in a software package, allowing to model data generating processes under complex non‐proportional hazard scenarios, to simulate data from these models and to perform the weighted logrank tests.

## 1 INTRODUCTION

Comparing survival distributions based on censored data is in general challenging since conclusions are sought about distributions which are observed incompletely. In medical statistics, and in particular in oncology, the logrank test and the Cox proportional hazards model have been established as key tools. Under the assumption of proportional hazards, they are efficient and their results can be interpreted unambiguously. The proportional hazards assumption implies that the benefit gained through active treatment over control is the same at each time‐point. Even though this assumption may be reasonable in a homogeneous population and over a limited time span, it is increasingly challenged in more complex settings.^1^

Here we investigate four different sources of non‐proportional hazards which are frequently encountered in oncology trials with survival endpoints. We address (1) the effect of delayed onset of treatment action, which has been described particularly for the new class of immuno‐oncology drugs.^2^ We take into account (2) the possibility of reduced survival probabilities after a disease progression event. This is a source of non‐proportional hazards if, for example, the active treatment prolongs the time to progression in addition to reducing the hazard rate in each disease state. We further study (3) the impact of differential effect of the treatment in different subgroups. This is of particular interest if stronger effects are expected in patients who are positive for a certain biomarker. For example, in metastatic colorectal cancer, targeted therapy inhibiting the epidermal growth factor receptor is effective only against tumors that are free from mutations in the KRAS or NRAS genes.^3^ Subgroup effects have also been described in settings where a fraction of patients is permanently cured (see for example, the examples in Reference 4).

Finally (4), in an actual trial patients may at some time‐point receive medication that is different from the planned medication in their trial arm, for various reasons. One focus of the paper is on treatment switching where patients from the control group may receive the potentially active treatment after they have experienced disease progression, or in an alternative scenario, patients from either group may receive an effective follow‐up medication after disease progression. For example, in a randomized controlled trial in metastatic non‐small‐cell lung cancer, 33% of patients in the control group received the treatment group medication after disease progression.^5^ In the standard intention‐to‐treat analysis non‐proportional hazards arise since the observed treatment effect is reduced with time as a consequence of treatment switching. Alternative methods to analyze data in presence of treatment switching been studied extensively. In particular, accelerated failure time models accounting for the time under control and active treatment have been proposed.^6^, ^7^ Our focus is on investigating the effect of switching in an intention‐to‐treat analysis.

When comparing two hazard functions which are not proportional, the power of the logrank test can be restored by weighting observed events. Optimal weights would correspond to the actual event time specific log hazard‐ratio,^8^ which is unknown. In practice, weighted logrank tests emphasizing either early, intermediate or late event times via an appropriate weighting function may be used to accommodate for an expected pattern of non‐proportionality.

In this manuscript, we focus on the Fleming‐Harrington *ρ* − *γ* family of weighted logrank tests.^9^ Since the choice of a powerful weighting function is subject to prior assumptions, we further consider the properties of maximum‐type tests^9^, ^10^, ^11^ that combine a set of differently weighted logrank test.

To study the operating characteristics of these tests under the aforementioned sources of non‐proportional hazards, we propose a data generating model based on mixtures of survival distributions that are defined via piecewise constant hazards. Similar methods have been applied in the literature to study the sole impact of delayed onset,^12^ of a subpopulation of cured patients^4^, ^13^ and the combined effect of delayed onset, disease progression and treatment switching, though not subgroups of patients.^14^ However, subgroups with different response characteristics are an important element to be considered in trials for targeted therapies such as immuno‐oncology drugs. Even in studies with restrictive inclusion criteria, the study population may consist of previously unknown subpopulations.

Furthermore, the recruitment scheme and trial duration have an impact on the distribution of observed event times. Under non‐proportional hazards, the power of hypothesis tests is also depending on this distribution, see for example, Reference 13. Consequently, we also study the influence of different recruitment rates under the considered non‐proportional hazards scenarios.

In the manuscript, we aim to provide guidance on the methods for the primary analysis under differently complex non‐proportional hazard settings. To this end we further provide software as an R package labeled nph^15^ which allows one to model data generating processes under complex non‐proportional hazard scenarios, to simulate data from these models and to analyze datasets using the weighted logrank tests we studied.

The remainder of the manuscript is structured as follows. In Section 2 the proposed data generating model is defined. In Section 3 the weighted logrank tests are described. In Section 4 we present a case study based on an actual trial.^5^ In Section 5 the power of weighted logrank and maximum tests depending on different sources of non‐proportional hazards and their extents is investigated systematically by simulation. Details on R package nph ^15^ providing functions to simulate and analyze data under non‐proportional hazards are given Section 6. We conclude with a discussion in Section 7. Additional information on the simulation scenarios and results for further simulation scenarios are provided in the online supplemental material.

## 2 MODELING NON‐PROPORTIONAL HAZARDS

Let subscripts *i* ∈ {*ctr*, *trt*} denote the control and treatment group in a randomized controlled clinical trial with survival as main endpoint. For a patient in group *i*, let the survival time *T* ∈ [0, ∞) be a continuous non‐negative random variable with distribution function *F*_*i*_(*t*), survival function *S*_*i*_(*t*) = 1 − *F*_*i*_(*t*) and density *f*_*i*_(*t*). The hazard function is defined as *λ*_*i*_(*t*) = lim_*h* ↓ 0_*P*(*T* ∈ (*t*, *t* + *h*] ∣ *T* > *t*)/*h* = *f*_*i*_(*t*)/*S*_*i*_(*t*). The cumulative hazard is defined as $\Lambda_{i}\left(t\right)=\int_{0}^{t}\lambda_{i}\left(s\right)\mathrm{ds}$. The survival function can be expressed as *S*_*i*_(*t*) = exp(−Λ_*i*_(*t*)).

**FIGURE 1:**
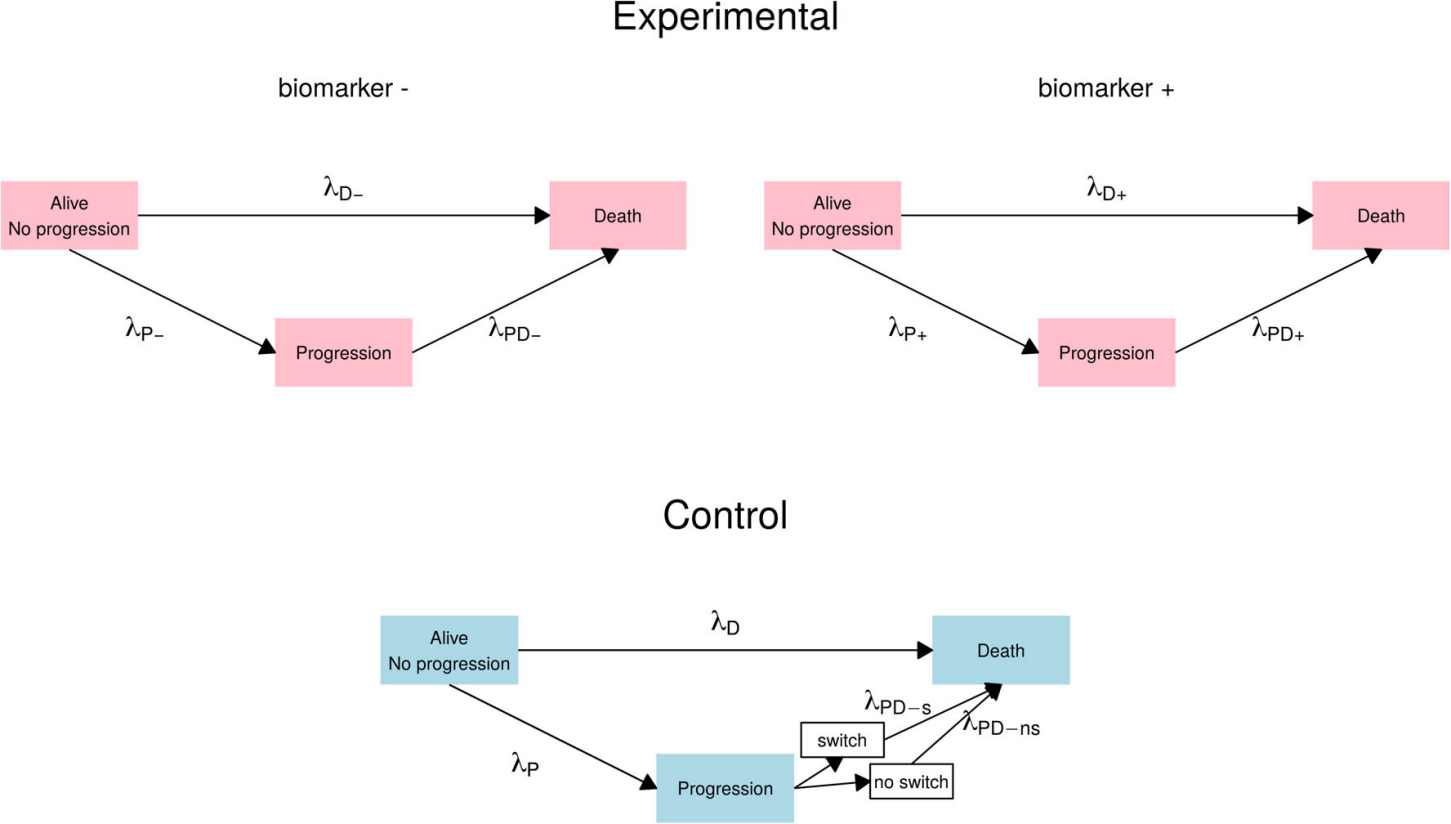
Multi‐state representation of the modeled sources of non‐proportional hazards. Different states are represented by boxes, transitions are represented by arrows. The transition rates are indicated next to the respective arrows. All transition rates may be piecewise constant functions of time. For simplicity of representation, biomarker dependent subgroups are shown only in the experimental group and treatments switching is only indicated in the control group. However the proposed framework allows for both, subgroups and treatment switching, in each group

For traditional study planning, the survival functions are often modeled via constant hazards over time, resulting in exponentially distributed survival times. Such a model may fail to address the complexity of actual trial data and in particular, does not cover non‐proportional hazards. In this section, we propose a model that is still simple and easy to apply but allows for increasingly complex hazard functions and in particular addresses several sources of non‐proportional hazards. Figure 1 illustrates the different states and rates assumed in this model.

### 2.1 Piecewise constant hazards and delayed onset of treatment effect

The hazard rates *λ*_*i*_(*t*), *i* ∈ {*trt*, *ctr*} may be modeled as piecewise constant functions to provide an intuitively simple model with non‐proportional hazards that still allows for complexity when needed.

Define *k* time intervals [*t*_*j* − 1_, *t*_*j*_), *j* = 1,…, *k* with 0 = *t*_0_ < *t*_1_ < *t*_*k*_ = ∞ and constant hazards *λ*_*ij*_. The resulting hazard function in group *i* is $\lambda_{i}\left(t\right)=\sum_{j=1}^{k}\lambda_{\mathrm{ij}}1_{t\in \left[t_{j-1},t_{j}\right)}$, the cumulative hazard function is $\Lambda_{i}\left(t\right)=\int_{0}^{t}\lambda_{i}\left(s\right)\mathrm{ds}=\sum_{j=1}^{k}\left(\left(t_{j}-t_{j-1}\right)\lambda_{\mathrm{ij}}1_{t>t_{j}}+\left(t-t_{j-1}\right)\lambda_{\mathrm{ij}}1_{t\in \left[t_{j-1},t_{j}\right)}\right)$ and we obtain the survival function as $S_{i}\left(t\right)=e^{-\Lambda_{i}\left(t\right)}$.

In particular, we apply the piecewise constant hazard approach to model the effect of delayed onset of treatment action. Assume that the treatment has an effect on the hazard rate only after a certain time span *t*_*onset*_ from initiation of the treatment. The hazard rate in the treatment group is modeled as $\lambda_{\mathrm{trt}}\left(t\right)=\lambda_{\text{preonset}}1_{t<t_{\text{onset}}}+\lambda_{\text{postonset}}1_{t\geq t_{\text{onset}}}$ whereas the hazard rate in the control group is constantly *λ*_*ctr*_(*t*) = *λ*_*preonset*_. The resulting survival curves are identical until time *t*_*onset*_ and are separating afterward (assuming *λ*_*postonset*_ is different from *λ*_*preonset*_).

A similar model has been used by Hasegawa^12^ to provide a sample size formula for weighted logrank tests under the scenario of a delayed treatment effect. While we focus on models used for study planning, piecewise constant hazard models may also be estimated given observed data, see for example, References 16, 17.

### 2.2 Changing hazards after disease progression

The piecewise constant hazard functions defined above allow for changing hazards at defined time‐points. However, the hazard rate may also change at random time‐points, in particular after progression of disease (PD). To model this aspect, we assume that the time to disease progression *T*_*PD*_ is governed by a separate process with hazard function *η*_*i*_(*t*), *i* ∈ {*trt*, *ctr*}, which does not depend on the hazard function for death *λ*_*i*_(*t*). *η*_*i*_(*t*), too, may be modeled as piecewise constant or, for simplicity, as constant over time. Define *λ*_*i*,*prePD*_(*t*) and *λ*_*i*,*postPD*_(*t*) as the hazard functions for death before and after disease progression. Conditional on *T*_*PD*_ = *s*, the hazard function for death in group *i* is *λ*_*i*_(*t*| *T*_*PD*_ = *s*) = *λ*_*i*,*prePD*_(*t*)*I*_*t* ≤ *s*_ + *λ*_*i*,*postPD*_(*t*)*I*_*t* > *s*_ and the conditional survival function is $S_{i}\left(t|T_{\mathrm{PD}}=s\right)=\exp\left(-\int_{0}^{t}\lambda_{i}\left(t|T_{\mathrm{PD}}=s\right)\mathrm{ds}\right)$. The unconditional survival function results from integration over all possible progression times as $S_{i}\left(t\right)=\int_{0}^{t}S_{i}\left(t|T_{\mathrm{PD}}=s\right)\mathrm{dP}\left(T_{\mathrm{PD}}=s\right)$. Note that, even though *η*_*i*_(*t*) does not depend on *λ*_*i*_(*t*), progression free survival and overall survival times will be correlated since progression free survival time is min(*T*, *T*_*PD*_).

### 2.3 Biomarker subgroups

A further element in our model are subgroups in the patient population that may exhibit different hazard functions. Patients who are positive with respect to certain biomarkers may show a better response to treatment. For modeling differential subgroup effects, we assume the number and relative sizes of subgroups are known, however in the analysis we regard the presence of subgroups as an unknown aspect. Thus, we are interested in the marginal survival functions over the full population. Given *m* subgroups with relative sizes *p*_1_, …, *p*_*m*_ and subgroup‐specific survival functions *S*_*i*,*l*_(*t*), the marginal survival function is the mixture $S_{i}\left(t\right)=\sum_{l=1}^{m}p_{l}S_{i,l}\left(t\right)$. Note that the respective hazard function is not a linear combination of the subgroup‐specific hazard functions. It may be calculated by the general relation $\lambda_{i}\left(t\right)=-\frac{{\mathrm{dS}}_{i}\left(t\right)}{\mathrm{dt}}\frac{1}{S_{i}\left(t\right)}$.

Other authors have focused on the presence of a subgroup of long‐term survivors or completely cured patients and their implications in study planning,^4^, ^13^ sample size reassessment^18^ and data analysis.^19^

### 2.4 Treatment switching after progression

The final aspect in our model is the possibility that the study medication is changed, for example, to a further line treatment, due to a disease progression event. Such treatment switching may occur with a certain probability only. We address the probabilistic aspect by defining two subpopulations, nested within any subpopulation as defined previously, of patients that will switch medication after disease progression and patients that will not switch with survival functions *S*_*l*,*switch*_(*t*) and *S*_*l*,*noswitch*_(*t*). Denote the relative sizes of these two subpopulations as *p*_*l*,*switch*_ and *p*_*l*,*noswitch*_, such that *p*_*l*,*switch*_ + *p*_*l*,*noswitch*_ = *p*_*l*_ and *p*_*l*,*switch*_/*p*_*l*_ corresponds to the probability for switching in subgroup *l*. The marginal survival function, covering subpopulations and switching, is $S_{i}\left(t\right)=\sum_{l=1}^{m}\sum_{s\in \left\{\text{switch},\text{noswitch}\right\}}p_{l,s}S_{i,l,s}\left(t\right)$. This approach is a specific application of a scenario with changing hazards after disease progression as considered in Section 2.2.

### 2.5 Sampling from the modeled survival function

Once the marginal survival functions *S*_*i*_(*t*), *i* ∈ {*ctr*, *trt*} are derived, we may draw random samples via the inverse c.d.f. method. That is, draw a uniform random number *U* ∼ *Unif*(0, 1) and calculate the random survival time as $S_{i}^{-1}\left(U\right)$. In the numeric calculations we regard *t* as discrete with smallest increments of 1 day, which facilitates the computation of the inverse function.

Note that sampling *n* observations from the overall marginal distribution of the population is equivalent to first sampling the subgroup status of *n* patients and then sampling the conditional survival times given the subgroups.

## 3 HYPOTHESIS TESTING

We consider the setting of randomized clinical trials comparing an experimental treatment to a control treatment. The aim of such trials typically is to show superiority of the experimental treatment over control. Under a proportional hazards assumption the treatment effect is conveniently expressed as hazard ratio which facilitates formulating an appropriate null hypothesis and deciding on a testing procedure. Typically, to avoid any further assumptions on the underlying distribution of survival times, the logrank test or, equivalently, a test based on the Cox proportional hazard model is applied, which is an optimal strategy in this context.^20^

Under non‐proportional hazards (in combination with censored data) deciding on a relevant parametrisation of the testing problem is less straight forward. In this paper we focus on the general one‐sided null hypothesis of (weighted) logrank tests, which is *H*_0_ : *λ*_*ctr*_(*t*) ≤ *λ*_*trt*_(*t*), ∀ *t* ≥ 0. Rejecting *H*_0_ means there is a treatment benefit at least in some time interval. See Section 7 for further discussion on this approach and on alternative effect measures.

### 3.1 (Weighted) logrank tests

The logrank test is frequently applied to test the one‐sided null hypothesis *H*_0_ : *λ*_*ctr*_(*t*) ≤ *λ*_*trt*_(*t*), ∀ *t* ≥ 0. Note that *H*_0_ implies *S*_*ctr*_(*t*) ≥ *S*_*trt*_(*t*), ∀ *t* ≥ 0. The reverse implication *S*_*ctr*_(*t*) ≥ *S*_*trt*_(*t*), ∀ *t* ≥ 0 ⇒ *λ*_*ctr*_(*t*) ≤ *λ*_*trt*_(*t*), ∀ *t* ≥ 0 is true under proportional hazards but does not generally hold under non‐proportional hazards (see^8^).

For a given sample, let D be the set of unique event times. For a time‐point t∈D, let *n*_*t*,*ctr*_ and *n*_*t*,*trt*_ be the number of patients at risk in the control and treatment group and let *d*_*t*,*ctr*_ and *d*_*t*,*trt*_ be the respective number of events. The expected number of events in the control group is calculated under the least favorable configuration in *H*_0_, *λ*_*ctr*_(*t*) = *λ*_*trt*_(*t*), as $e_{t,\mathrm{ctr}}=\left(d_{t,\mathrm{ctr}}+d_{t,\mathrm{trt}}\right)\frac{n_{t0}}{n_{t0}+n_{t1}}$. The conditional variance of *d*_*t*,*ctr*_ is calculated from a hypergeometric distribution as $\mathrm{var}\left(d_{t,\mathrm{ctr}}\right)=\frac{n_{t0}n_{t1}\left(d_{t0}+d_{t1}\right)\left(n_{t0}+n_{t1}-d_{t0}-d_{t1}\right)}{{\left(n_{t0}+n_{t1}\right)}^{2}\left(n_{t0}+n_{t1}-1\right)}$. Further define a weighting function *w*(*t*). The weighted logrank test statistic for a comparison of two groups is

$$z=\sum_{t\in D}w\left(t\right)\left(d_{t,\mathrm{ctr}}-e_{t,\mathrm{ctr}}\right)/\sqrt{\sum_{t\in D}w{\left(t\right)}^{2}\mathrm{var}\left(d_{t,\mathrm{ctr}}\right)}$$

Under the least favorable configuration in *H*_0_, the test statistic is asymptotically standard normally distributed and large values of *z* are in favor of the alternative.

Under proportional hazards and for local alternatives (ie, the hazard ratio approaching 1), the unweighted logrank test is the most powerful rank‐invariant test for *H*_0_.^20^ With arbitrary hazard functions, optimal weights for testing *H*_0_ would correspond to the true log‐hazard ratio *w*(*t*) = log(*λ*_*ctr*_(*t*)/*λ*_*trt*_(*t*)) when *λ*_*ctr*_(*t*) > *λ*_*trt*_(*t*) and *w*(*t*) = 0 otherwise.^8^ The true hazard function is of course unknown. However, by defining particular weight functions *w*(*t*) the effect size at early or late event times may receive greater influence on the test statistic.

In this paper we consider particular weights in the Fleming‐Harrington *ρ* − *γ* family^9^ $w\left(t\right)=\hat{S}{\left(t\right)}^{\rho }{\left(1-\hat{S}\left(t\right)\right)}^{\gamma }$. Here, $\hat{S}\left(t\right)=\prod_{s\in D:s\leq t}1-\frac{d_{t,\mathrm{ctr}}+d_{t,\mathrm{trt}}}{n_{t,\mathrm{ctr}}+n_{t,\mathrm{trt}}}$ is the pooled sample Kaplan‐Meier estimator. Weights *ρ* = 0, *γ* = 0 correspond to the standard logrank test with constant weights *w*(*t*) = 1. Choosing *ρ* = 0, *γ* = 1 puts more weight on late events, *ρ* = 1, *γ* = 0 puts more weight on early events and *ρ* = 1, *γ* = 1 puts most weight on events at intermediate time points.

### 3.2 Maximum logrank test

Depending on the true unknown alternative, differently weighted logrank tests may differ substantially in power. In general hypothesis testing problems, instead of deciding a‐priori for a single test, a viable strategy is to combine different tests for the same hypothesis via the mean or maximum of their individual test statistics.^21^ In many applied problems, including weighted logrank tests, the asymptotic joint distribution of test statistics is multivariate normal, which facilitates the derivation of an approximate distribution of a combination statistic. In this context Tarone^10^ studied the distribution of the maximum of the unweighted logrank test statistic and the generalized Wilcoxon statistic which uses as weights the total number at risk at each event time. Lee^11^ compared the operating characteristics of combination tests based on the maximum or an average of *ρ* = 0, *γ* = 1 and *ρ* = 1, *γ* = 0 weighted logrank test statistics. The simulation results of Lee suggest that the maximum was the more robust combination statistic in terms of preserving power across various scenarios. Li et al^22^ studied the performance of the same statistics and several other test statistics, which are based on integrated differences of estimates of the survival functions or cumulative hazard functions, under scenarios of crossing survival functions. The robustness of the maximum test was confirmed also in this setting.

Karrison^23^ describes the implementation of a maximum‐type logrank test based on different *ρ* − *γ* weights for the software Stata. Recently, the idea to apply maximum‐type combination tests to analyze survival data with non‐proportional hazards is gaining increasing attention.^24^

To perform a maximum‐type combination test, a set of *r* different weight functions *w*_1_(*t*),…, *w*_*r*_(*t*) is specified and the correspondingly weighted logrank statistics *z*_1_,…, *z*_*r*_ are calculated. The maximum test statistic is *z*_*max*_ = max_*i* = 1, *r*_*z*_*i*_. If at least one of the selected weight functions results in high power, we may expect a large value of *z*_*max*_. Under the least favorable configuration in *H*_0_, approximately (*Z*_1_,  *Z*_*r*_) ∼ *N*_*r*_(**0**, **∑**). The *P*‐value of the maximum test, $P_{H_{0}}\left(Z_{\max}>z_{\max}\right)=1-P\left(Z_{1}\leq z_{\max},Z_{\text{r}}\leq z_{\max}\right)$, is calculated based on this multivariate normal approximation via numeric integration.

This approach automatically corrects for multiple testing with different weights and does so efficiently since the correlation between the different tests is incorporated in **∑**. For actual calculations, **∑** is replaced by an estimate.

Note that *cov*(*w*_*i*_(*t*)*d*_*t*,*ctr*_, *w*_*j*_(*t*)*d*_*t*,*ctr*_) = *w*_*i*_(*t*)*w*_*j*_(*t*)*var*(*d*_*t*,*ctr*_), at least approximately assuming weights are converging in probability to a non‐random function. Thus the *i*, *j*th element of **∑** is estimated as

$$c\hat{o}v\left(Z_{i},Z_{j}\right)=\sum_{t\in D}w_{i}\left(t\right)w_{j}\left(t\right)\mathrm{var}\left(d_{t,\mathrm{ctr}}\right)/\sqrt{\sum_{t\in D}w_{i}^{2}\left(t\right)\mathrm{var}\left(d_{t,\mathrm{ctr}}\right)\sum_{t\in D}w_{j}^{2}\left(t\right)\mathrm{var}\left(d_{t,\mathrm{ctr}}\right)}$$

Since the individual tests are typically highly correlated, the required multiplicity adjustment can be moderate while there is a good chance to gain from the most powerful among the included weighted test.

## 4 CASE STUDY

To investigate increasingly complex examples for a trial under non‐proportional hazards we devised five case study scenarios. As example for an actual trial which shared several characteristics of these scenarios see Reference 5. For easier interpretation, piecewise constant hazard functions are described by the median event time corresponding to the respective hazard rate in each time interval, that is, the presented median event time for interval *i* equals *log*(2)/*λ*_*i*_.

### 4.1 Scenario A

As reference, we assume a scenario with proportional hazards, with the hazard for death corresponding to median survival times of 20 months and 12 months for the treatment and control arm, respectively.

### 4.2 Scenario B

For the treatment arm we assume a hazard for death before PD corresponding to a median survival time of 24 months, a hazard for death after PD corresponding to a median survival time of 16 months, and a hazard for PD corresponding to 12 months median time to progression. For the control arm, the respective hazards correspond to median times of 22, 7 and 7 months, for the hazard of death before and after PD and for the hazard of PD, respectively.

### 4.3 Scenario C

In this scenario we assume a delayed effect. During the first 2 months, the treatment group has the same hazards as the control group. After the first 2 months, the hazards used in scenario B for the treatment group are used.

### 4.4 Scenario D

We consider delayed effect and the subgroup of female patients (prev 50%) with an additional benefit. During the first 2 months, the treatment group has the same hazards as the control group (irrespective of subgroups). After 2 months, and for the subgroup of male patients, we keep the true underlying parameters as in Scenario B. For the subgroup of female patients, we assume no disease progression and a hazard for death corresponding to a median time of 24 months is used.

### 4.5 Scenario E

Here we consider treatment switching after disease progression for patients under the control treatment. Building on the previous scenarios, now we assume that 1/3 of the patients in the control group switch to the treatment group after progression. Note that for these patients, we also account for female/male subgroups with a prevalence of 50% each.

### 4.6 Scenario F

Finally, we study a scenario where treatment switching to an effective follow‐up medication occurs in both groups after disease progression. Similar to scenarios A and B, we assume that before PD the hazard for death corresponds to a median of 20 and 12 months, in the treatment and control group respectively, and the rates for PD correspond to median times of 12 and 7 months. After progression, patients in either group are switched to an effective follow‐up medication with 75% probability, and the subsequent hazard for death corresponds to 18 months. For patients who are not switched we assume subsequent median survival times of 12 and 7 months.

**FIGURE 2:**
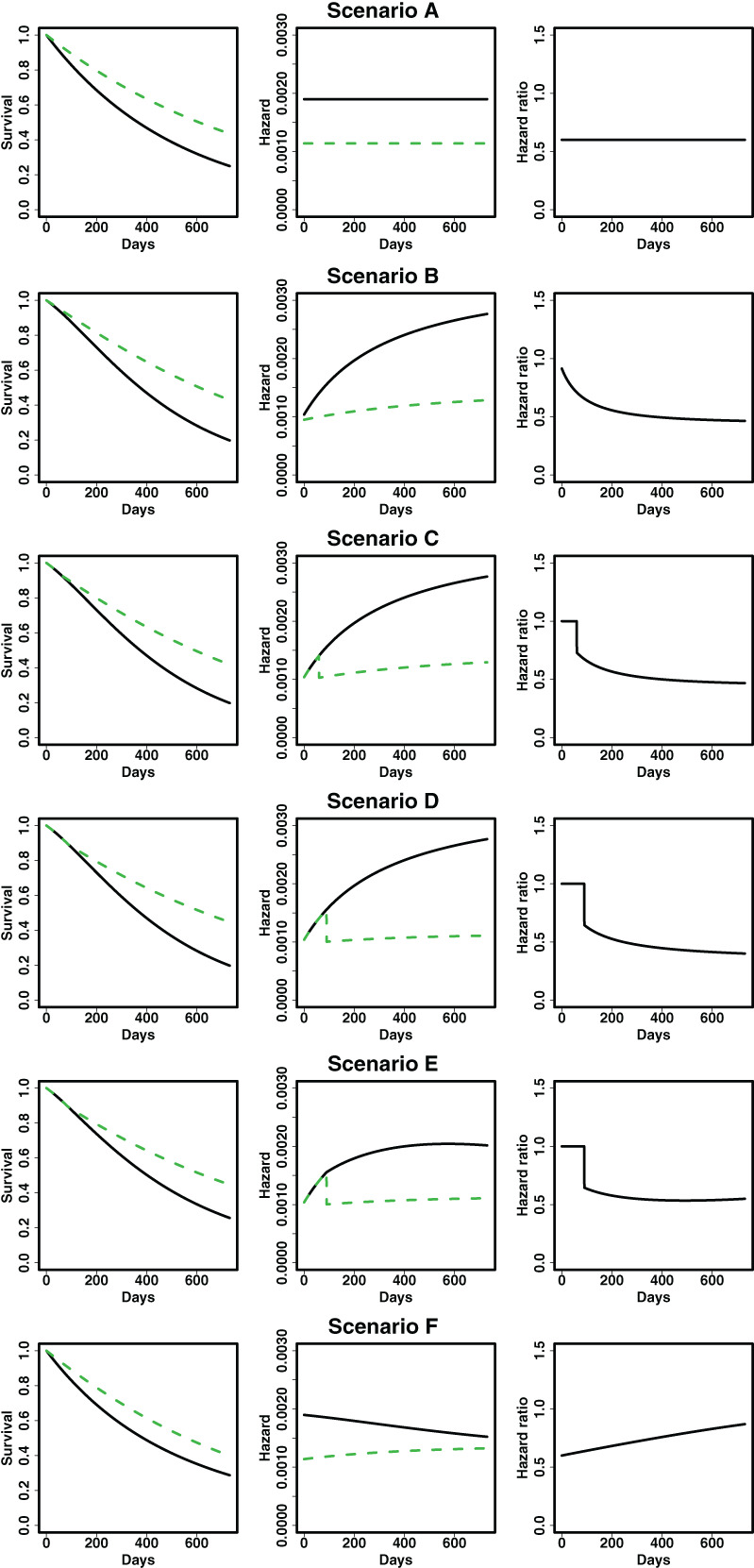
Survival function, hazard function and hazard ratio for treatment (dash green line) and control (solid black line) arms as described in Scenarios A‐F

We present the theoretical survival, hazard and hazard ratio functions over time in Figure 2. An interesting aspect is that the survival curves are similar across the scenarios, even if we compare the case where the proportionality assumption holds with the cases where it does not. In an actual trial, the investigator will only examine a single realization from the theoretical survival function and, therefore, it may not be easy to identify non‐proportionality from the observed curves.

Table 1 shows the power for the weighted logrank test and the maximum test across the four scenarios under design assumptions similar to Reference 5. We assumed that patients are recruited over 1 year with a constant rate of 616 patients per year and are randomized between the treatment and control arm with a 2:1 allocation ratio.

**TABLE 1:** Power of weighted logrank tests in the studied scenarios for weighted logrank tests with (*ρ*, *γ*) ∈ {(0, 0), (0, 1), (1, 1), (1, 0)}, and a maximum test combining all four logrank tests (denoted by Max log‐rank 4). The weighted logrank tests with (*ρ* = 0, *γ* = 0) corresponds to the conventional logrank test

			Scenario					
Test	*ρ*	*γ*	A	B	C	D	E	F
Max logrank 4			91.8	92.4	86.1	89.3	77.1	78.1
Weighted logrank	0	0	93.0	90.9	78.8	78.1	65.3	80.9
Weighted logrank	0	1	82.4	91.1	87.3	91.3	79.8	57.5
Weighted logrank	1	1	85.9	92.7	88.3	91.4	80.6	63.8
Weighted logrank	1	0	92.6	87.8	71.8	69.3	57.0	81.7

The study ends when 190 events have been observed. This value corresponds to 80% of the number of events observed in Reference 5 and was chosen such that the power of the considered tests in our case study is approximately between 80% and 90%.

Censoring was included only in terms of administrative censoring at the end of the study. The one‐sided level of significance was 0.025. The power was calculated from 50 000 simulation runs. As expected, the usual logrank test (*ρ* = *γ* = 0) outperforms the others in Scenario A since the proportional hazards assumption holds. However, in Scenarios C, D and E, using the weighted logrank test with stronger weights on late events provides higher power and in Scenario F stronger weights on early events provides higher power. In this sense, the maximum logrank test performs the best, since its power is among the highest in all cases.

## 5 SIMULATION STUDIES

In this section we evaluate the effect of the different sources of non‐proportional hazards on the power of unweighted logrank tests, the *ρ* − *γ* weighted logrank tests with (*ρ*, *γ*) ∈ {(0, 1), (1, 1), (1, 0)}, and two maximum‐type tests. The first maximum test combines all four logrank tests, the second one combines the unweighted logrank test and the test with weights *ρ* = 0, *γ* = 1, which puts more weight in late events and should be beneficial under delayed onset.

We perform simulations for three study designs corresponding to slow, intermediate and fast recruitment. In all simulation scenarios patients are randomly assigned to treatment or control with equal probabilities. The recruitment periods are 2 years, 1 year and half a year for the scenarios of slow, intermediate and fast recruitment, with constant recruitment rates of 100, 300 and 800 patients per year, respectively. In the simulations, trials stop for data analysis when a total number of 130 events is reached. (Under proportional hazards this number of events would correspond to approximately 80% power of the logrank test with a hazard ratio of 0.6 at one‐sided level of significance of 0.025.) To focus on the effects of non‐proportional hazards we did not consider random censoring due to drop outs in the simulation. However, the R package nph^15^ we provide does allow for additional random censoring.

Data are simulated from survival functions that were defined using the methods described in Section 2 and with parameters detailed below in this section. All tests are performed at a one‐sided level of significance of 0.025. For each scenario we performed 50 000 simulation runs.

Details on the survival functions and hazard functions in the considered scenarios can be found in Supplemental material S1.

### 5.1 Effect of subgroup prevalence

Here we assume a subgroup of patients has increased benefit from treatment. We modeled patients under control have a constant hazard corresponding to median survival of 11 months, and patients under treatment to have constant hazards with 18 months median survival if they do not belong to the subgroup and 30 months median survival if they do. The prevalence of the subgroup was varied in (0,0.2,0.4,0.6). Not that these are population prevalences, with observed sample proportions deviating according to random sampling.

In a second set of scenarios we added a delay of 100 days before onset of the treatment effect, meaning that in the first 100 days the hazard rate under treatment is the same as under control (for both subgroups) and afterward corresponds to the values of the previous scenario.

**FIGURE 3:**
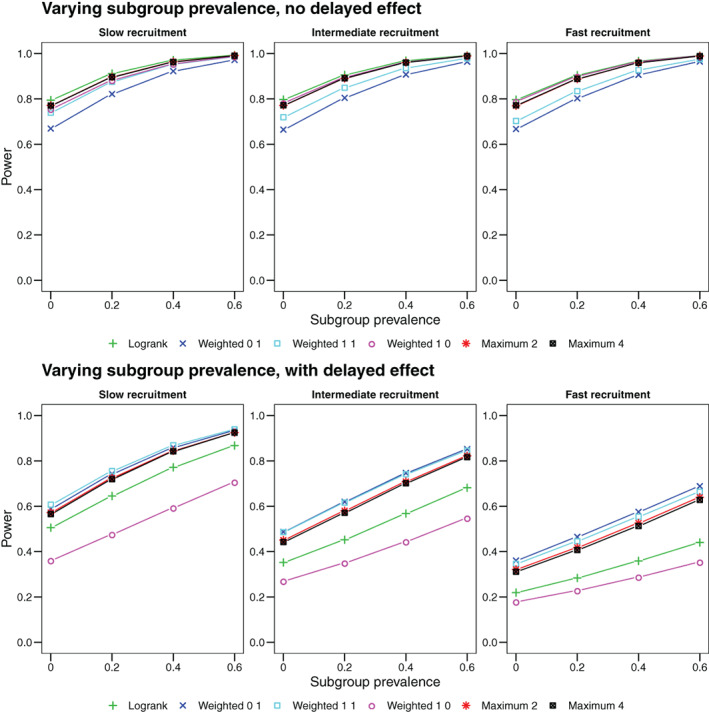
Power as a function of subgroup prevalence. Upper row: Scenarios without delayed onset of treatment effect. Lower row: Scenarios with delayed onset of treatment effect after 100 days. We show the power for the logrank test and three weighted logrank tests with (*ρ*, *γ*) ∈ {(0, 1), (1, 1), (1, 0)} and maximum tests (i) combining the logrank and (*ρ* = 0, *γ* = 1) (denoted by Maximum 2) and (ii) combining all four weighted logrank tests (denoted by Maximum 4)

Visualizations of the resulting survival and hazard functions are included in the online supplemental material. Figure 3 shows the resulting power curves. Without delayed onset the extent of non‐proportionality of hazard rates is limited (see Supplement), hence the logrank test is superior to the other tests. Also there is little dependence on the recruitment scheme. In scenarios with delayed effect, however, there is a strong non‐proportionality and, both, putting too much weight on early events as well as recruiting too fast and hence observing mainly early events results in reduced power. Both maximum tests perform similar and the drop in power when using either maximum test compared to the best single weighted logrank test is small.

### 5.2 Effect of hazard ratio in subgroup

**FIGURE 4:**
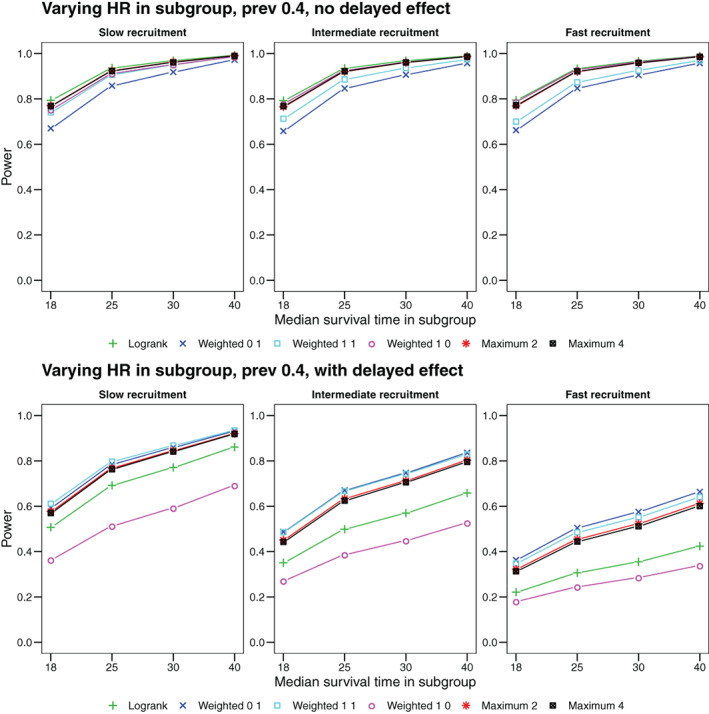
Power as a function of the additional hazard ratio in a subgroup with 40% prevalence. Upper row: Scenarios without delayed onset of treatment effect. Lower row: Scenarios with delayed onset of treatment effect after 100 days. We show the power for the logrank test and three weighted logrank tests with (*ρ*, *γ*) ∈ {(0, 1), (1, 1), (1, 0)} and maximum tests (i) combining the logrank and (*ρ* = 0, *γ* = 1) (denoted by Maximum 2) and (ii) combining all four weighted logrank tests (denoted by Maximum 4)

Here we assume a setup identical to Section 5.1, except that the subgroup prevalence is held constant at 40% whereas the extra benefit in the subgroup is varying in terms of a multiplicative factor of the base hazard ratio in {1,0.72,0.6,0.45}, which corresponds to median survival times in the subgroup under treatment of {18,25,30,40}. As before, we considered an additional set of scenarios with delayed onset of treatment effect of 100 days. Resulting survival and hazard functions are found in the supplement and resulting power curves are shown in Figure 4. The observed pattern of power values is similar to that of the scenarios with varying subgroup prevalence, with increasing treatment benefit in the subgroup having a similar effect as increasing the subgroup prevalence.

### 5.3 Effect of treatment switching

We again start from a setup with hazard rates as in Section 5.1 and the subgroup prevalence held constant at 40%. We now add the possibility to switch from control to active treatment after disease progression. Progression is modeled as an independent process with constant hazard and median time to progression of 5 months (fast progression scenarios) or 9 months (slow progression scenarios). In the treatment group progression has no further effect. In the control, groups patients will switch to the treatment group with a probability varying between scenarios in {0,0.2,0.4,0.6}. After switching, former control patients will have the same hazards as a patient in the treatment group. The beneficial subgroup effect will come into effect for a switcher with a probability of 40%, since belonging to the subgroup is modeled as an intrinsic property of each patient. To calculate the power we assume an intention‐to‐treat analysis, that is, each patient is counted according to the initial randomization and with the full available observation time, regardless of potential subsequent switching.

**FIGURE 5:**
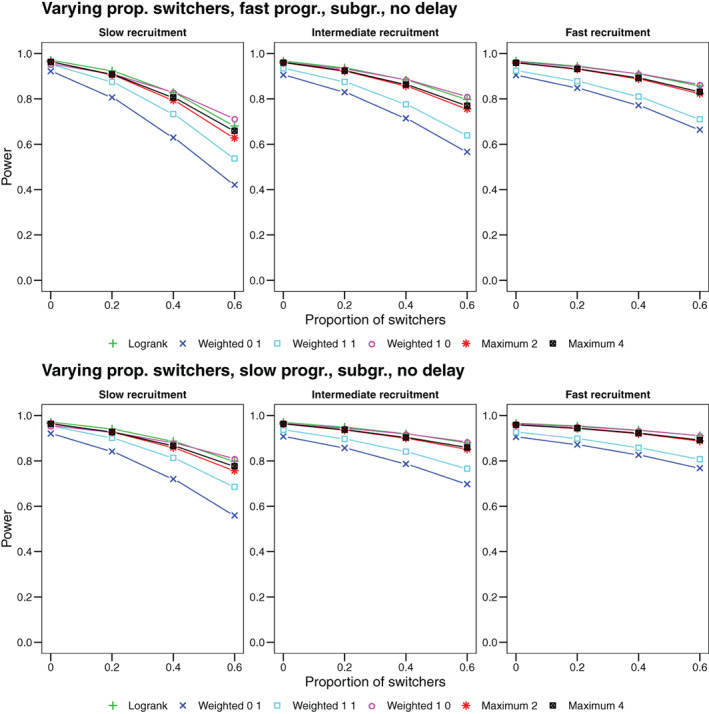
Power as a function of the probability to switch from control to treatment after disease progression. Upper row: Fast progression scenarios with a median time to progression of 5 months. Lower row: Slow progression scenarios with a median time to progression of 9 months. We show the power for the logrank test and three weighted logrank tests with (*ρ*, *γ*) ∈ {(0, 1), (1, 1), (1, 0)} and maximum tests (i) combining the logrank and (*ρ* = 0, *γ* = 1) (denoted by Maximum 2) and (ii) combining all four weighted logrank tests (denoted by Maximum 4)

As before, survival and hazard functions are shown in the supplement. Resulting power curves are shown in Figure 5. As the possibility of switching decreases the hazard ratio with time, tests with more weight on early events have more power and the loss in power when using less optimal weights can be substantial. As seen before, the maximum tests are able to recover most of the power of the best test they include. The maximum test that includes only the *ρ* = 0, *γ* = 0 and the *ρ* = 0, *γ* = 1 weighted tests is less powerful than the maximum test that includes all four considered weighted logrank tests. However, the difference is only up to few percentage points, since the unweighted logrank test still has considerably power if the effect is mostly present at early event times. When switching is the main cause of non‐proportional hazards, fast recruitment, and hence observing more early events, results in larger power values for all considered test, since in this scenario the observed treatment effect is then strongest at early event times. Similarly, the reduction in power with increasing switching probability is less pronounced in a fast recruitment regimen.

### 5.4 Effect of disease progression

**FIGURE 6:**
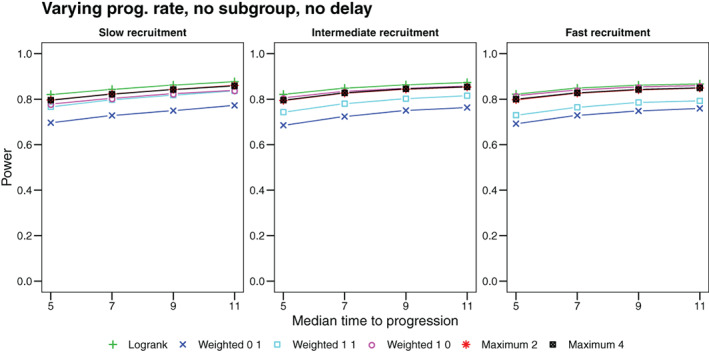
Power as a function of the median time to disease progression when hazards increase after progression in both groups. We show the power for the logrank test and three weighted logrank tests with (*ρ*, *γ*) ∈ {(0, 1), (1, 1), (1, 0)} and maximum tests (i) combining the logrank and (*ρ* = 0, *γ* = 1) (denoted by Maximum 2) and (ii) combining all four weighted logrank tests (denoted by Maximum 4)

We finally study the impact of altered hazard rates after disease progression. This results in non‐proportional hazards for death if disease progression is occurring at a different rate in the two groups, or if the between‐group hazard ratio is different before and after progression. Here we consider the former case. We assume constant hazard rates before and after progression, respectively, corresponding to median survival times of 18.33 and 15 months under treatment and 11 and 9 months under control. Hence the hazard ratio is 0.6, both, before and after progression. The rate of progression is constant with median time to progression of 5 months in the control group and a value in {5,7,9,11} months under treatment. Slower progression under treatment results in an initial decrease (stronger effect) in the hazard ratio over approximately 200 days and subsequent increase, however the extent of this non‐proportionality is rather small (see Supplement). Consequently, the logrank test is most powerful for this setting, closely followed by maximum tests and the *ρ* = 1, *γ* = 0 weighted test. The recruitment scheme does not have a notable impact in these tests, however, the power of other weighted tests, which emphasize late events, are affected by too fast recruitment. As expected, a stronger treatment effect with respect to delaying progression increases the power in all scenarios. Figure 6 shows the detailed power curves.

### 5.5 Summary of simulation results

The extent of non‐proportionality caused by different sources of non‐proportional hazard varies strongly. In the studied scenarios, delayed onset and the possibility of treatment switching resulted in the strongest deviations from proportional hazards. However, under more extreme scenarios also the presence of subgroups with different response to treatment and progression effects have the potential for more pronounced non‐proportionality. Delayed onset and treatment switching have opposing effects with respect to the time points of strongest observable effects, hence weighting early vs late events has opposing effects on power with these two sources on non‐proportionality. See Table 2 for a concise overview of the conclusions drawn from the simulation studies and literature. In line with previous simulation studies,^11^, ^22^, ^23^ the maximum test has the potential to safeguard against misspecification of a single weighed test. Interestingly, the price of combining two or four tests in the maximum test, in terms of reduced power compared to the best test single test in the simulation, was almost identical. This can be attributed to the strong correlation between the included tests and supports the idea of using the maximum tests as robust variant in the analysis of trials where the pattern of non‐proportional hazards cannot be predicted well.

**TABLE 2:** Summary of the effect of considered population characteristics and design aspects on the proportionality of hazards and the power of between group comparisons of survival times

Aspect	Effect on non‐proportional hazards or on power	Key message
Delayed onset of treatment effect	Treatment effect increases with time, since the treatment is effective only with some delay.	Reducing the weight of early events increases power. Zero weight for early events may be considered as most extreme case.^19^
Disease progression	Treatment effect changes with time if active treatment delays disease progression or the treatment effect is different before and after progression.	Optimal weights depend on treatment effect on progression and hazard ratios before and after progression. The effects can be explored by simulation in the proposed piecewise constant hazards framework.
Biomarker subgroups	Treatment effect is increasing with time, when a biomarker positive subgroup with particularly strong treatment benefit is included, since this subpopulation is enriched with time.	More weight on late events increases power. As an alternative to weighted logrank test, parametric cure rate models may be considered.^4^
Treatment switch after progression	Treatment effect is decreasing with time, if patients may switch to a common medication after disease progression.	More weight on early events may increase power. Accelerated failure time models accounting for different failure rates before and after treatment switch have been proposed as parametric alternative.^6^, ^7^
Recruitment scheme	Fast recruitment with short follow up results in larger power if the treatment effect decreases with time. Slow recruitment with long follow up results in larger power if the treatment effect increases with time.	Under non‐proportional hazards, the power of logrank tests also depends on the timing of observed events. This in turn depends on recruitment speed and total study duration. The effect of different recruitment schemes should be considered during study planning.

## 6 SOFTWARE IMPLEMENTATION

In Section 5 we studied the effect of different sources of non‐proportional hazards separately, while Section 4 provided a case study of a clinical trial in which at least two sources were present. Naturally, different diseases and trial characteristics will exhibit different patterns in terms of non‐proportionality mechanisms. We provide the R package nph ^15^ which is available at the CRAN repository so that researchers can explore different scenarios using the theory presented in this paper. The package includes functions to model survival distributions in terms of piecewise constant hazards and with differential effects due to disease progression and subgroups, to simulate data from the specified distributions, and to perform the weighted logrank tests and maximum tests described in Section 3.1.

Functions for modeling/setting the underlying survival model include:

- pchaz: Calculate survival functions defined through piecewise constant hazards
- subpop_pchaz: Calculate survival functions defined through piecewise constant hazards, allowing for a change in the piecewise constant hazard regimen after a random progression time
- pop_pchaz: Calculate survival functions for a mixture of subpopulations. In each subpopulation the survival function is defined through piecewise constant hazards and these are allowed to change after a random progression time.

Functions for generating simulated datasets given a specified survival model are:

- sample_fun: Sample survival times based on study settings
- sample_conditional_fun: Sample conditional survival times based on study settings and given observed interim data

Functions for performing statistical tests:

- logrank.test: Weighted logrank test
- logrank.maxtest: Maximum logrank test

Plotting functions:

- plot.mixpch: Generic plot function for mixpch objects, which result from pchaz, subpop_pchaz and pop_pchaz
- plot_diagram: Creates the diagram for the model, similar to Figure 1
- plot_shhr: Plot of survival, hazard and hazard ratio of two groups as function of time

Additionally, we provide in the supplementary material (S2 file) a document with basic usage instructions for the package and outlining further more complex examples of non‐proportional hazards along with the R code to model these situations.

## 7 DISCUSSION

The proportional hazards assumption may be violated due to different inherent features of a study design, patient population or mode of treatment action. The modeling approach pursued in this paper allows one to study the effect of different sources of non‐proportional hazards on the survival and hazard functions. It also allows for easy simulation of study data and comparison of different testing strategies under complex non‐proportional hazard scenarios.

Under proportional hazards the standard unweighted logrank test corresponds to the optimal test in the family of weighted logrank tests, whereas under non‐proportional hazards other weighting schemes may result in larger power. If the shape of the hazards function for an actual trial is well understood, for example, through modeling the assumed data generating process, and well known from available data, a suitable weighting function may be selected. For example, Liu et al^25^ proposed nearly optimal weights under a specific cure rate model with delayed onset. In many cases, however, different hazard functions may be considered reasonable. In this situation the combination of several weighting functions in terms of the maximum test may be preferable. The power of this test is typically closely below that of the best included individual test, providing a robust method. Other combination functions with similar properties may be explored though, for example, the combination of individual tests via the harmonic mean of their *P*‐values.^26^

Group sequential methods have been proposed as another approach to tackle the planning uncertainties under unknown patterns of non‐proportional hazards. Jimenez et al^27^ describe sample size reassessment methods in the setting of the delayed onset and comparing two groups with *ρ* − *γ* weighted logrank test. They propose to plan the sample size under a model of delayed onset, for example, for a *ρ* = 0, *γ* = 1 weighted test. If, however, the assumption about the delay was not correct, the sample size might be too small and in this case sample size reassessment based on interim is proposed to increase the sample size (and power) if necessary. Korn and Freidlin^28^ study the loss in power when futility stopping rules at interim are applied in the setting of delayed onset. To reduce the influence of the suspected delayed onset phase, they propose to modify stopping rules by the additional condition that a reasonable fraction (eg, at least two‐thirds) of the events observed at interim have occurred at least a sufficient time (eg, 3 months) after randomization. In the presence of a potential subgroup of long term survivors, the planned number of events may be reached later than anticipated. In this context, Chen^4^ proposed to select and fit a parametric model, choosing from a set of distributions such as lognormal and Weibull, with blinded interim data and thereon predict the remaining study duration.

We focused on testing the null hypothesis of ordered hazard functions. Here rejecting *H*_0_ using a (weighted) logrank test first of all means that at least for *t* in some time interval *λ*_*trt*_(*t*) < *λ*_*ctr*_(*t*). In the clinical trial setting this means the experimental treatment has some beneficial effect at least in some time interval. However, which time intervals are actually observed and how strongly their information contributes to the test decision depends on the censoring distribution and the applied weights (see Reference 8). A definite assessment of the treatment benefit should therefore include considerations on possible detrimental effects in time intervals which were not observed or which received less weight. In some cases subject matter knowledge on the safety of the treatment may even allow for the assumption *λ*_*trt*_(*t*) ≤ *λ*_*ctr*_(*t*) for all *t*.

However the survival of two (or more) groups may also be compared in terms of other parameters. A simple strategy is so the called landmark or milestone analysis in which the values of the survival functions for a pre‐specified time point, for example, one‐year survival, are compared between groups. However, selecting a fixed time point in the planning phase is difficult and an inappropriate choice could lead to power loss. In addition, this approach suffers from information loss as only one point of the estimated survival curve is considered. Logan and Mo^19^ combine both approaches. They argue that under non‐proportional hazards and under potential crossing of survival curves, long term survival is a relevant outcome. They propose a (group sequential) test for the intersection null hypothesis of equal survival function at a pre‐specified time point *t*_0_ and equal hazard functions after *t*_0_. Alternative effect measures which we did not consider here include the difference in restricted mean survival times^29^ and the average hazard ratio.

A well‐known alternative, the difference in restricted mean survival times^29^ of two groups may be used as effect measure. Tests based on this parameter can be more powerful than the unweighted logrank test, however depending on the actual hazard functions and the chosen time point *t*_*restr*_ up to which the restricted means are calculated.^30^ The interpretation of this parameter is entirely focused on the hazards before *t*_*restr*_, as the difference in restricted mean survival times corresponds to the expected life time gained up to the time *t*_*restr*_. Note that under crossing survival functions, the probability to survive longer than time *t*_*restr*_ may be larger in one group while the restricted mean survival time up to time *t*_*restr*_ is larger in the other group.

Also the general non‐parametric effect measure *P*(*T*_*trt*_ > *T*_*ctr*_) may be utilized, which is referred to under various names such as relative effect or probabilistic index. In this context *T*_*trt*_ and *T*_*ctr*_ are the event times of two randomly chosen subjects under treatment and control, respectively. Under proportional hazards, the hazard ratio equals $\frac{P\left(T_{\mathrm{trt}}>T_{\mathrm{ctr}}\right)}{1-P\left(T_{\mathrm{trt}}>T_{\mathrm{ctr}}\right)}$, see for example, Reference 31. For non‐proportional hazard settings, several authors propose to use as effect measure an average hazard ratio^32^, ^33^, ^34^, ^35^ defined as $\frac{\int \lambda_{\mathrm{trt}}/\left(\lambda_{\mathrm{trt}}+\lambda_{\mathrm{ctr}}\right)\mathrm{dW}\left(t\right)}{\int \lambda_{\mathrm{ctr}}/\left(\lambda_{\mathrm{trt}}+\lambda_{\mathrm{ctr}}\right)\mathrm{dW}\left(t\right)}$, with *W*(*t*) a survival function that serves as weighting function. When choosing *W*(*t*) = *S*_*ctr*_(*t*)*S*_*trt*_(*t*), the average hazard ratio conveniently equals $\frac{P\left(T_{\mathrm{trt}}>T_{\mathrm{ctr}}\right)}{1-P\left(T_{\mathrm{trt}}>T_{\mathrm{ctr}}\right)}$. However other choices of *W*(*t*) may result in larger power depending on the true hazard functions. With censored data, the average hazard ratio needs to be computed in a truncated fashion up to some time *t*_*trun*_, which is within the time span of observed events. In this case it can be interpreted to compare the survival times *T*_*trt*_ and *T*_*ctr*_ conditional on at least one of the two event times being smaller than *t*_*trun*_.

Explicit modeling of non‐proportional hazards may be considered, see for example, the parametric approach in Reference 4 discussed above. However, to allow for unambiguous interpretation of observed effects in a parametric analysis model, the model must be predefined and should be supported by subsequent model diagnostics.

There is not a unique answer on how to address the impact of different disturbances of the proportional hazards assumptions. As key finding from our investigation with respect to choosing a hypothesis test we conclude that the maximum test has the potential to safeguard against deviations from optimal assumptions and is robust with respect to the number of subsumed tests. With respect to identification of sources non‐proportionality we conclude that inspection of estimated survival curves, for example, from previous trials, does in general not reveal underlying sources of non‐proportional hazards. In part as consequence of the difficulty to empirically identify these sources, we conclude that simulations are essential to understand the operational characteristics of a planned trial under all relevant deviations from the proportional hazards assumption.

The modeling approach, the hypothesis tests and the according software we described provide a comprehensive set of tools to address these challenges. While we focused on weighted logrank test for statistical inference on hazard functions, the set of method can readily be extended with analysis methods based on further parameters of interest.

## Supporting information


**Appendix S1** Supporting information

**Appendix S2** Supporting information

## Data Availability

"We provide the R package nph [15] which is available at the CRAN repository so that researchers can explore diffferent scenarios using the theory presented in this paper." [See Section 6]
