# Supplementary material for: Delayed treatment effects, treatment switching and heterogeneous patient populations: How to design and analyze RCTs in oncology
Source: Pharm Stat. 2020 Aug 23;20(1):129–45. doi: 10.1002/pst.2062 (PMC7818232; doi:10.1002/pst.2062)

Delayed treatment effects, treatment switching and heterogeneous patient populations: how to design and analyse RCTs in oncology
Supplementary material 1: Simulation scenarios

Robin Ristl, Nicolás M Ballarini, Heiko Götte, Armin Schüler, Martin Posch, Franz König (2020-04-16)

In this document we provide more details on the simulation scenarios. Consider a clinical trial in which patients are randomised to either an experimental treatment or a control one. The considered endpoint is survival time and the follow-up time is 6-years.

Effect of subgroup prevalence

Varying subgroup prevalence, no delayed effect

In this scenario, we evaluate the effect of the presence of a binary biomarker (+ or -) that defines a subgroup with differential treatment effect. This is illustrated in the following figure, where we show the hazard for a patient in the control group (black line), the hazard for a biomarker + patient in the treatment group (dotted blue line), and the hazard for a biomarker - patient in the treatment group (solid blue line). The hazards correpond to a median survival time of 11 months in the control arm and 30 and 18 months in the biomarker + and biomarker - groups in the treatment arm, respectively.

Hazards for individuals

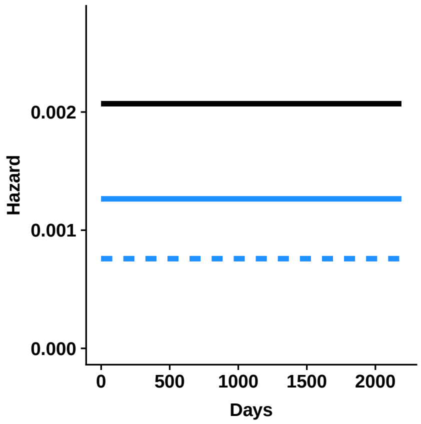

In the following figures we illustrate the survival and hazard curves for the population under study considering various subgroup prevalences. The solid black line corresponds to the curves in the control group while the dashed blue lines correspond to the curves in the treatment group. Additionally, the hazard ratio is displayed on the right, which allows visualizing the impact of the subgroup prevalence on the non-proportionality as the curve is not constant when there is a subgroup (prevalence not 0).

Population curves

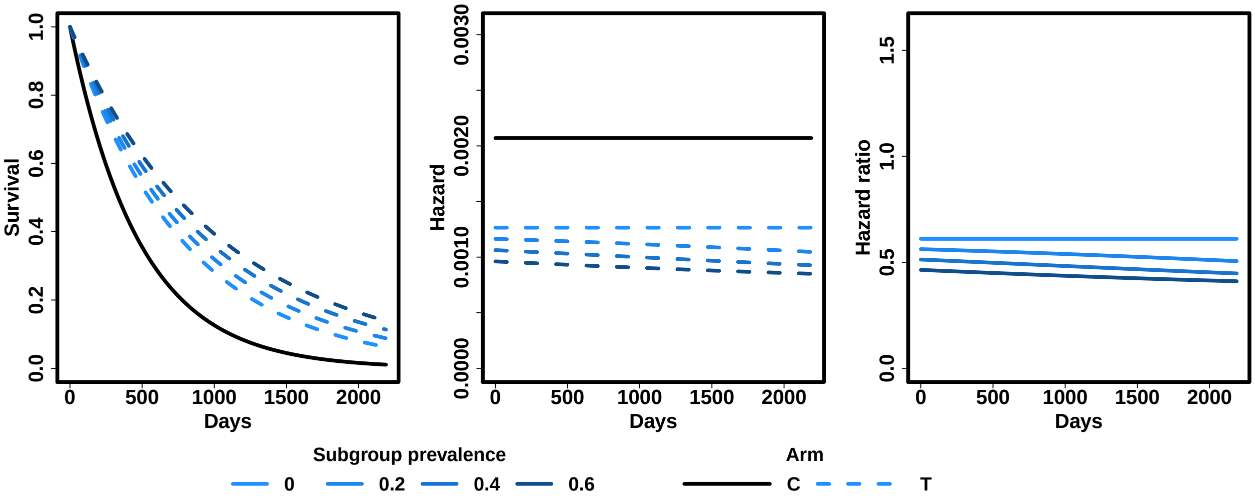

Varying subgroup prevalence, with delayed effect

In this scenario, we consider again the case of a presence of a binary biomarker, but we added an additional element: delayed response. This situation is illustrated in the figure below. The reduction in hazards in the treatment groups is only after 100 days of treatments.

Hazards for individuals

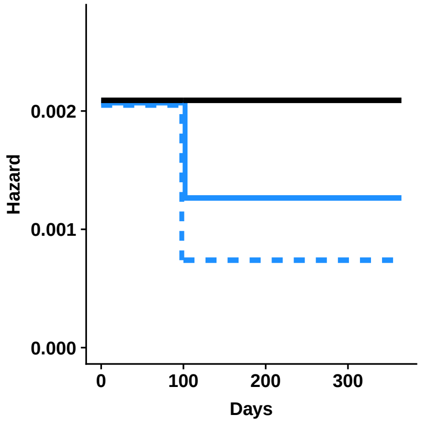

In the figures below, we show the population curves varying the subgroup prevalences.

Population curves

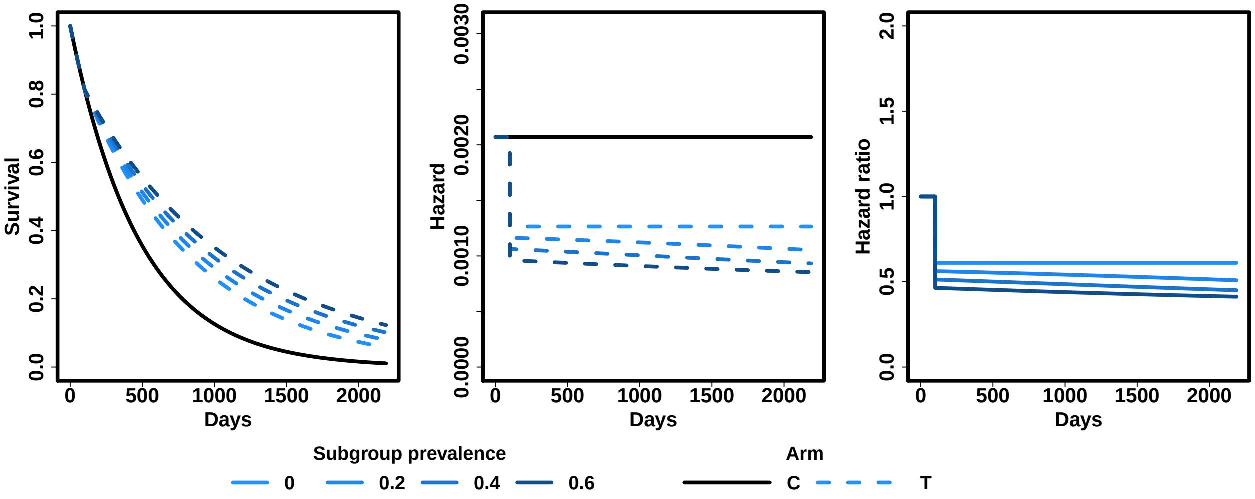

Effect of hazard ratio in subgroup

Varying HR in subgroup, prev 0.4, no delayed effect

In this scenario we evaluate the impact of different effects in the biomarker + subgroup. The subgroup prevalence is fixed at 0.4, but the hazard is varied so that the median survival time for the patients in the subgroup is 18, 25, 30, or 40 months. The median survival time in the treatment group for biomarker - patients is 18 and in the control group is 11. The figure below represents the hazards for a patients in the control group (solid black line), a biomarker - patient in the treatment group (solid blue line), and a biomarker + patient in the treatment group (dotted blue line).

Hazards for individuals

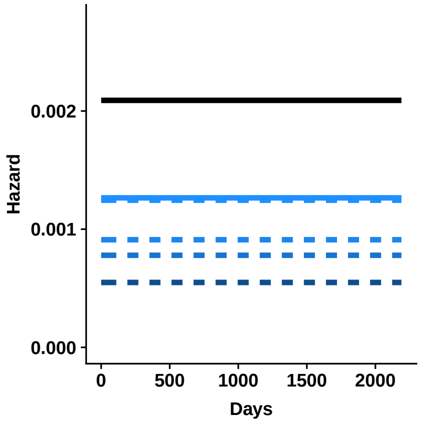

The impact of the different treatment effects in the biomarker + subgroup is illustrated in the figures below, where we again show the population survival and hazard curves, together with the hazard ratio.

Population curves

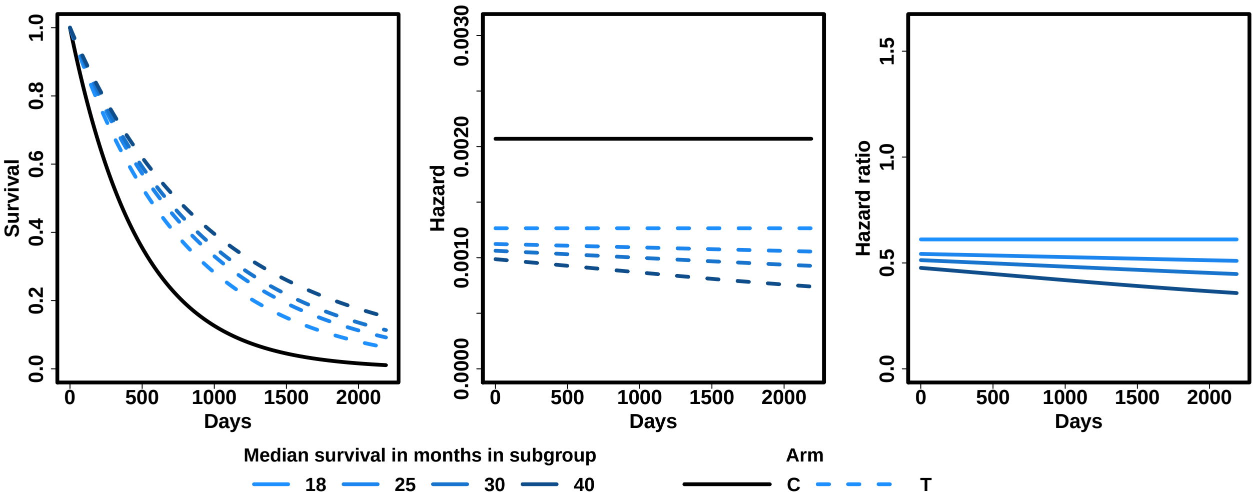

Varying HR in subgroup, prev 0.4, with delayed effect

In this scenario we add delayed response to the scenario 2a as done before:

Hazards for individuals

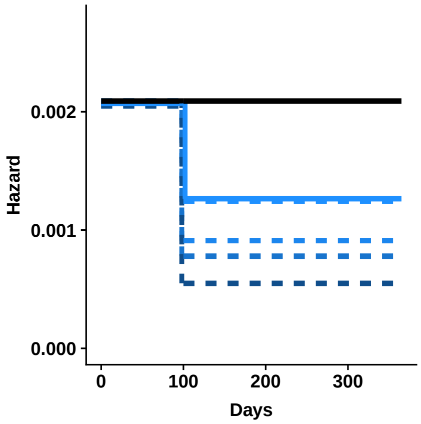

Population curves

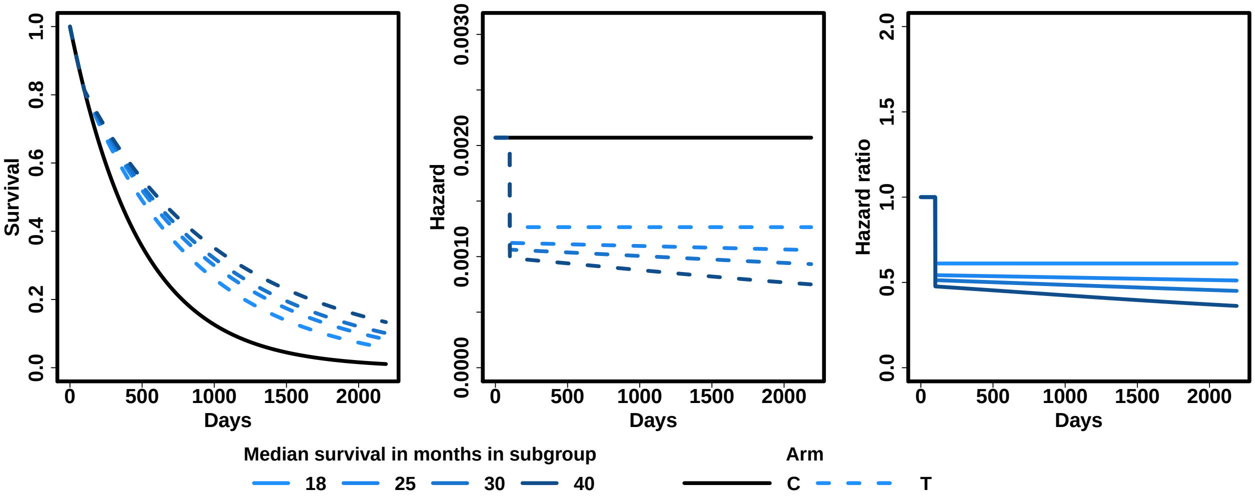

Effect of treatment switching

Varying prop. switchers, fast progr., subgr., no delay

In this scenario, we consider biomarker + and - subgroups, but we also consider as an additional element the case of disease progression and treatment switchers. In this situation, a proportion of the patients that are in the control group switch to the experimental treatment if they experience disease progression. To illustrate this, consider the figure below. The blue lines correspond to patients in the treatment group (solid: biomarker -, dash: biomarker +). The solid black line represents a patient in the control group that does not switch to the experimental treatment. The vertical line represents the median time to progression (at 5 months) for patients in the treatment group. However, the actual time of disease progression for an individual patient will not necessarily be exactly 5 months, but will be around this time. The black dashed line then represents a biomarker + patient in the treatment group that, after disease progression, is switched to the experimental treatment. The black long dashed line represents a biomarker - patient that after progression is switched to the experimental treatment.

Hazards for individuals

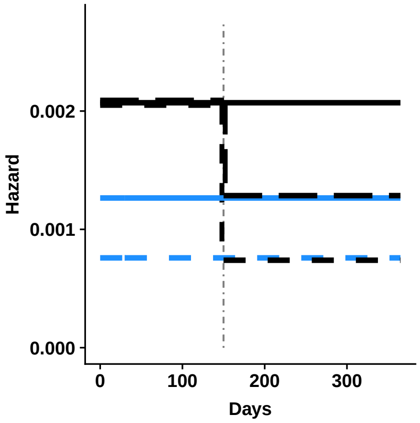

Following the ITT principle, patients randomized to the control group will be analized as patients in this arm even if they switch treatment. Therefore, the population curves for the control treatment will vary depending on the proportion of patients that switch treatment. Below we represent the population curves for four different proportions of switchers.

Population curves

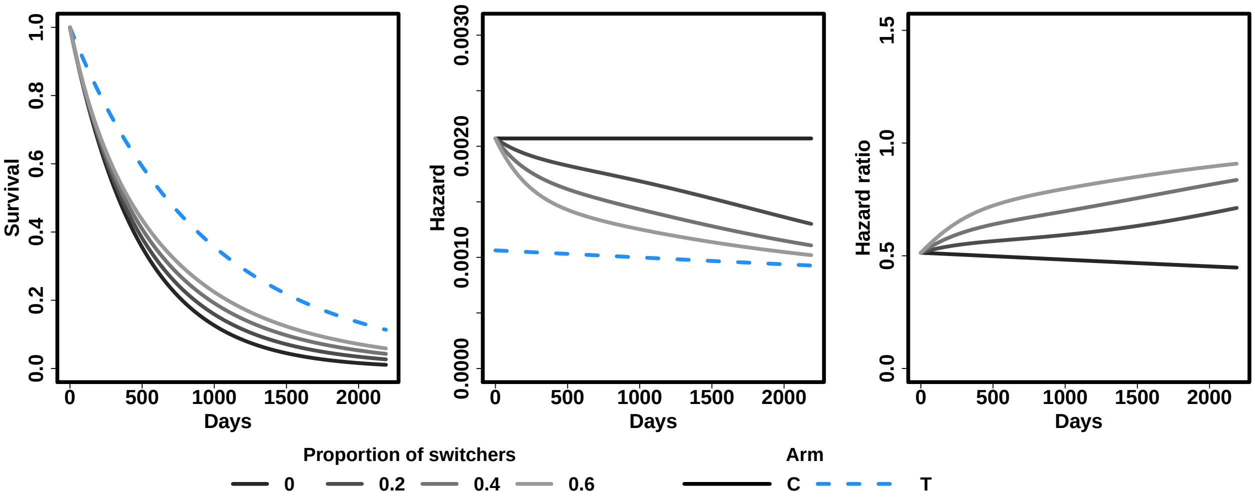

Varying prop. switchers, slow progr., subgr., no delay

This scenario is similar to the one illustrated above, 3a, but the median time to progression in the control group is 9 months instead of 5.

Hazards for individuals

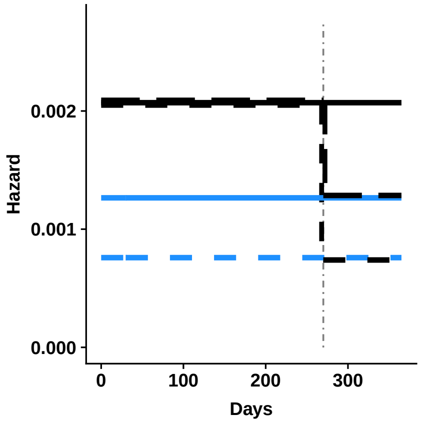

Following the ITT principle, patients randomized to the control group will be analized as patients in this arm even if they switch treatment. Therefore, the population curves for the control treatment will vary depending on the proportion of patients that switch treatment. Below we represent the population curves for four different proportions of switchers.

Population curves

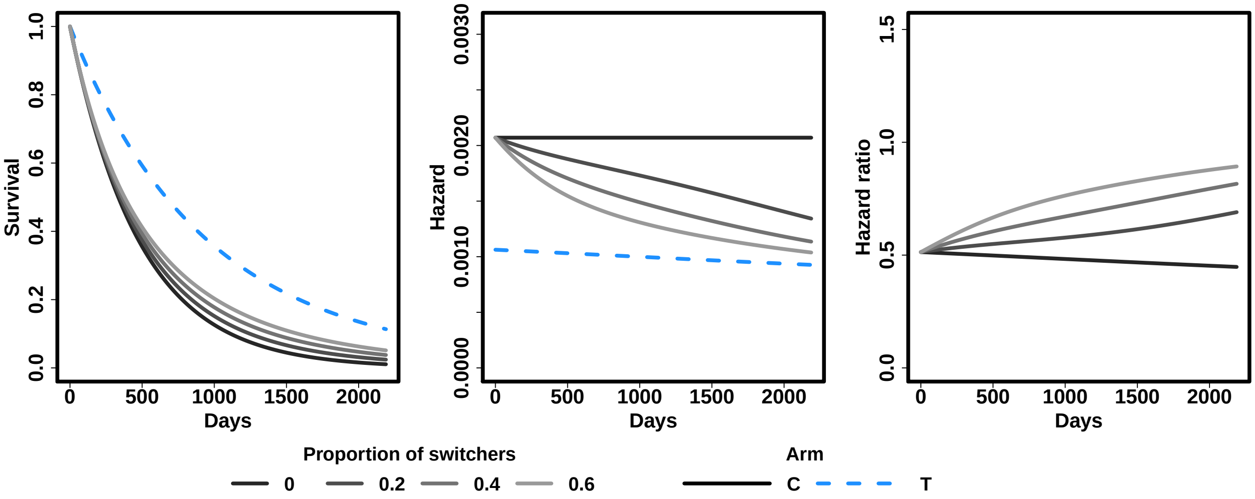

Effect of disease progression

Varying prog. rate, no subgroup, no delay

In this scenario, we evaluate the impact of different progression rates on patients. For the control group, we assume a median time progression of 5 months. After progression, the hazard for a patient in the control group is increased, lowering the median survival time grom 11 to 9 months. For patient in the treatment group, we assume four different situations varying the time to progression to be 5, 7, 9 and 11 months. For patients in the treatment group, the median time to death is 18.3 months before progression and 15 after. We do not assume biomarker + and - in this scenario.

Hazards for individuals

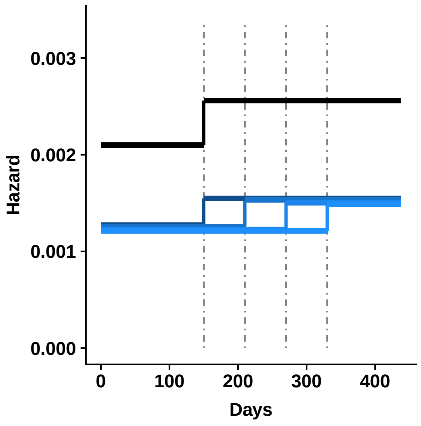

The effect of different progression times on the population curves and in the hazard ratio is illustrated in the figures below.

Population curves

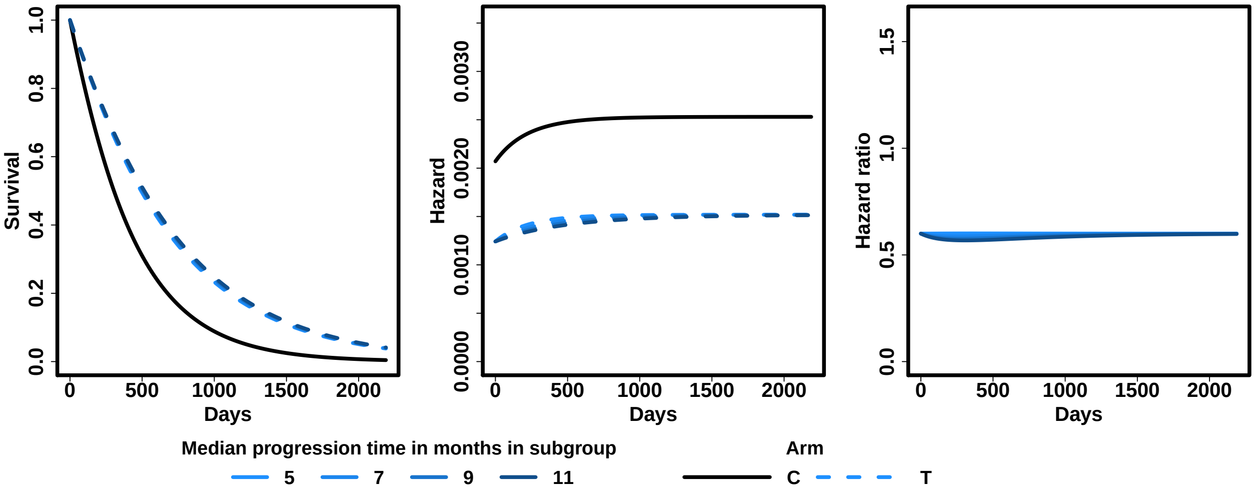

Supplement: Supplementary file 1 — Appendix S1 Supporting information [file PST-20-129-s001.pdf]
