# Supplementary material for: Delayed treatment effects, treatment switching and heterogeneous patient populations: How to design and analyze RCTs in oncology
Source: Pharm Stat. 2020 Aug 23;20(1):129–45. doi: 10.1002/pst.2062 (PMC7818232; doi:10.1002/pst.2062)
Supplement: Supplementary file 2 — Appendix S2 Supporting information [file PST-20-129-s002.pdf]

Delayed treatment effects, treatment switching and heterogeneous patient populations: how to design and analyse RCTs in oncology
Supplementary material 2: Introduction to nph, usage instructions and examples

Robin Ristl, Nicolás Ballarini, Heiko Götte, Armin Schüler, Martin Posch, Franz König

2020-04-16

Overview

The nph package includes functions to model survival distributions in terms of piecewise constant hazards and to simulate data from the specified distributions.

Installation

The package is available from CRAN and can be installed directly from R.

```
install.packages("nph")
```

Getting started

Basically, there are three mechanisms for non-proportionality available in this package:

- Disease progression
- Different effect by time intervals
- Subgroups

These scenarios are illustrated in the following figures. Note that the hazard ratio is not constant across time.

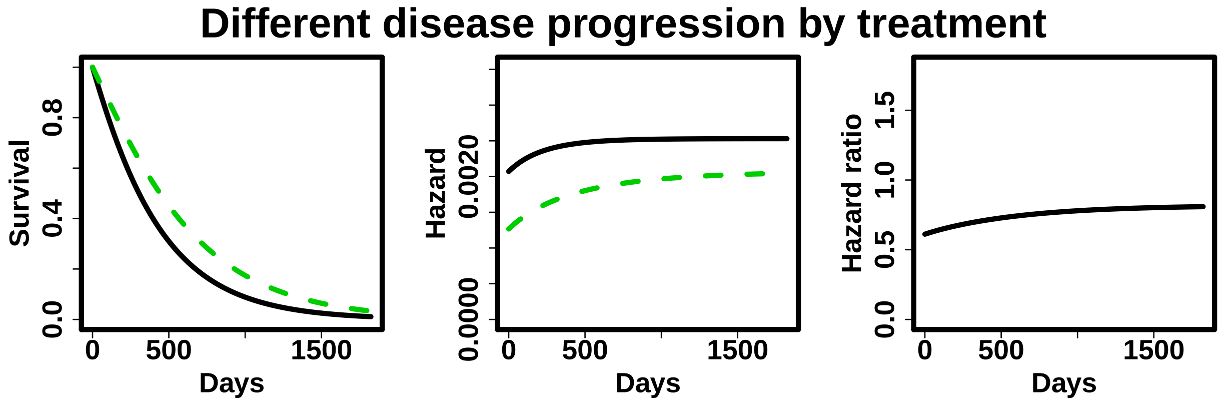

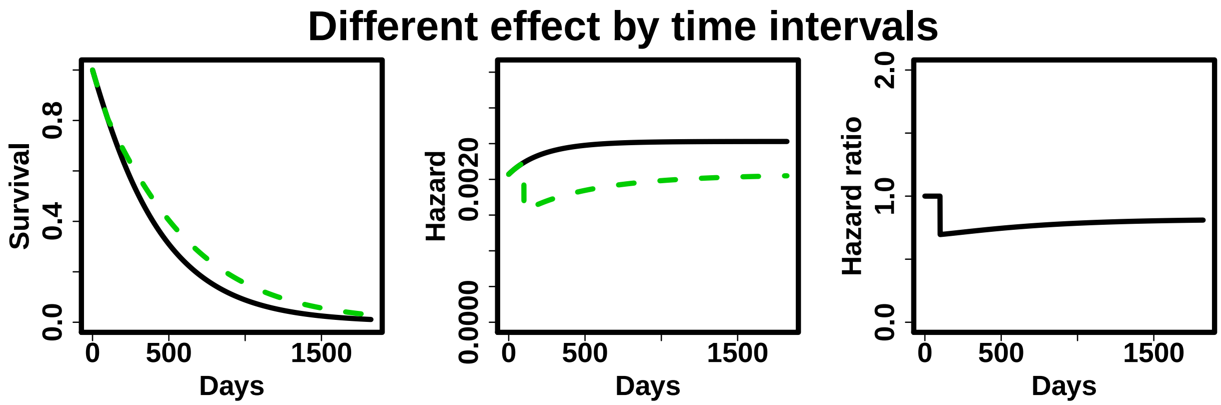

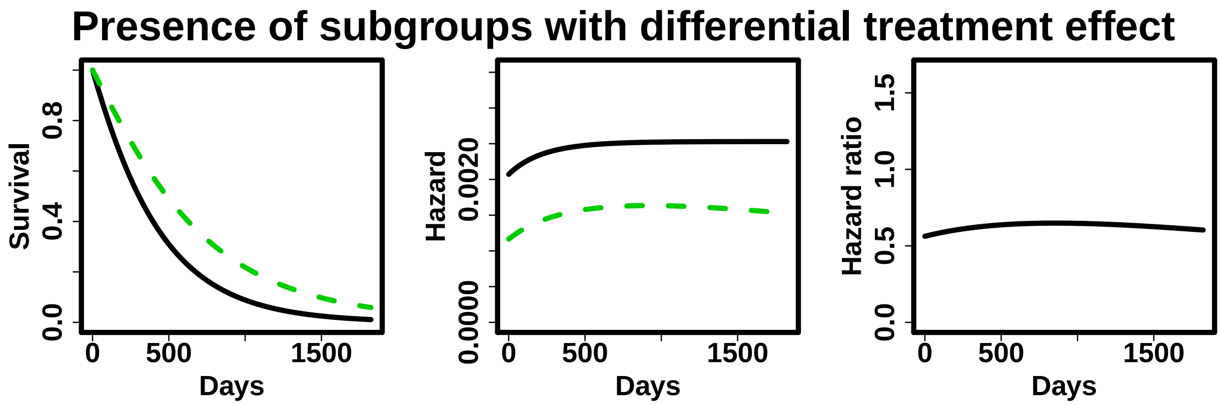

Basics

The functions of the package can be grouped according to their functionality.

Functions for modelling/setting the underlying survival model:

- `pchaz`
- `subpop_pchaz`
- `pop_pchaz`

Functions for generating simulated dataset given for a specified survival model:

- `sample_fun`
- `sample_conditional_fun`

Functions for performing statistical tests:

- `logrank.test`
- `logrank.maxtest`

Plotting functions:

- `plot.mixpch`

- plot_diagram
- plot_shhr

The basic underlying model for the survival mechanism assumes that each patient can be in one of three states: Alive with no progression of disease, Alive with progression of disease, and Dead.

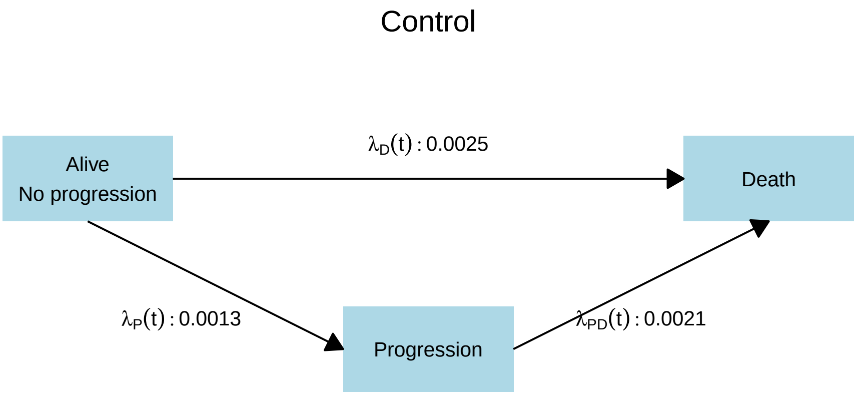

Creating the population model with pop_pchaz

The first step is to create the population model with the `pop_pchaz` function. As the previous figure shows, there are three hazard rates that need to be defined: the hazard of disease progression $\lambda_P(t)$, the hazard of death given no progression $\lambda_P(t)$, and the hazard of death given progression $\lambda_P(t)$. The arguments `lambdaProgMat`, `lambdaMat1`, and `lambdaMat2` in the `pop_pchaz` function correspond to the three hazard rates, respectively.

The hazard rates are assumed piecewise constant functions across k time intervals $[t_{j-1}, t_j)$, $j = 1, \ldots, k$ with $0 = t_0 < t_1 < \ldots < t_k = \infty$. Therefore, the `pop_pchaz` function has also an argument `T` that is a vector to specify $t_0, t_1, \ldots, t_k$. When `T` is of length 2 (and therefore only one time interval) `lambdaProgMat`, `lambdaMat1`, and `lambdaMat2` are scalars. If `T` is of length > 2, then the `lambdaProgMat`, `lambdaMat1`, and `lambdaMat2` are matrices where the number of columns is equal to the number of time intervals

$$\begin{bmatrix} \lambda^{[t_0 - t_1)} & \lambda^{[t_1 - t_2)} & \ldots & \lambda^{[t_{k-1} - t_k)} \end{bmatrix}.$$

For example, if the patients are followed for two years but the hazards change after the first year, then `T` should be specified as `c(0, 365, 2*365)`. If we assume a hazard rate for death of 0.02 and 0.04 for the first and second year respectively, the we should specify `lambdaMat1 = matrix(c(0.02, 0.04), ncol = 2)`.

Finally, is is also possible to specify different hazard rates for subgroups. The `pop_pchaz` has the argument `p` which is intended to specify the subgroup prevalences. Given m subgroups with relative sizes $p_1, p_2, \ldots, p_m$, then the `p` argument should be specified as `c(p_1, p_2, ..., p_m)`. The `lambdaProgMat`, `lambdaMat1`, and `lambdaMat2` then should have the number of row equal to the number of defined subgroups:

$$\begin{bmatrix} \lambda_1 \\ \lambda_2 \\ \ldots \\ \lambda_m \end{bmatrix}.$$

For example, if patients can be divided into two subgroup with prevalences 0.2 and 0.8 with hazard rates a hazard rate for death of 0.02 and 0.03 thoughout a one year interval, then we define `T = c(0, 365)`, `p = c(0.2, 0.8)` and `lambdaMat1 = matrix(c(0.02, 0.03), nrow = 2)`.

Naturally, it is possible to combine multiple time intervals and subgroups, then the hazard matrices have the form:

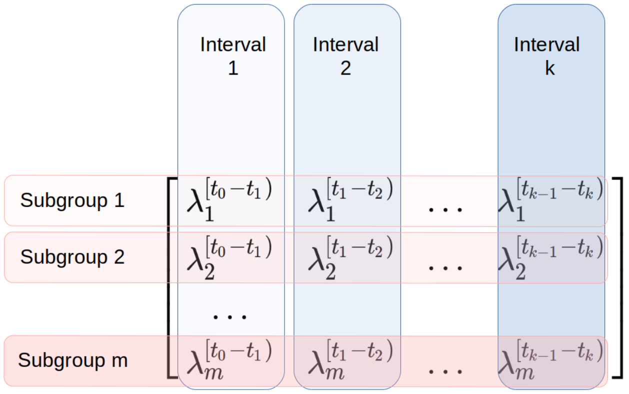

Below, we consider an example where there two subgroups and two time intervals. In practice, this situation correspond to the case where there is a delayed effect of the drug. Note that for specifying the hazard matrices, we use the median time to death/progression and use the function `m2r` (also provided in the package) to obtain the hazard rates.

```
times <- c(0, 100, 5 * 365)    # Time interval boundaries, in days
t_resp <- c(0.2, 0.8) #Proportion of subgroups
B5 <- pop_pchaz(
  T = times,
  lambdaMat1     = m2r(matrix(c(11, 30,
                               11, 18), byrow = TRUE, nrow = 2)),
  lambdaMat2     = m2r(matrix(c( 9, 20,
                                9, 11), byrow = TRUE, nrow = 2)),
  lambdaProgMat = m2r(matrix(c( 5, 15,
                                5,  9), byrow = TRUE, nrow = 2)),
  p = t_resp, discrete_approximation = TRUE
)
```

The results object is of class `mixpch`, which has a dedicated plotting function to visualize the survival and hazard functions.

```
plot(B5, main = "Survival function")
plot(B5, fun = "haz", main = "Hazard function")
plot(B5, fun = "cumhaz", main = "Cumulative Hazard function")
```

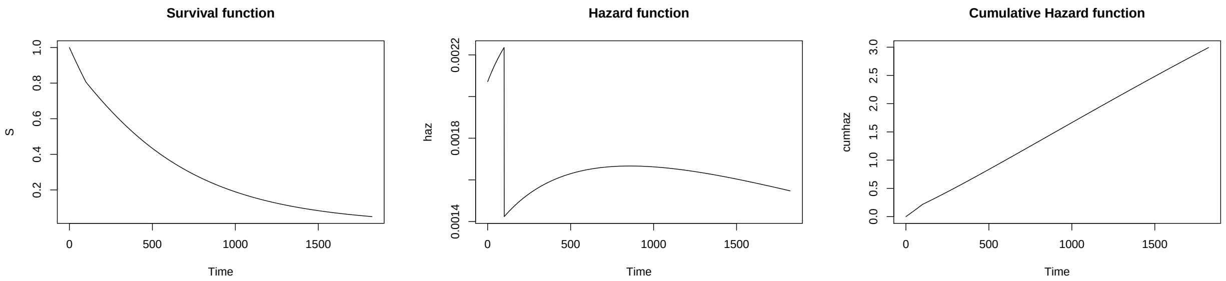
Survival function
S
1.0
0.8
0.6
0.4
0.2
0
500
1000
1500
Time
Hazard function
haz
0.0022
0.0018
0.0014
0
500
1000
1500
Time
Cumulative Hazard function
cumhaz
3.0
2.5
2.0
1.5
1.0
0.5
0.0
0
500
1000
1500
Time

Creating a simulated dataset with sample_fun

The sample_fun function is designed to generate a simulated dataset that would be obtained from a parallel group randomised clinical trial.

The first step is to create two objects with the (theoretical) survival functions for the treatment and control groups using `pop_pchaz`:

```
times <- c(0, 100, 5 * 365)   # Time interval boundaries, in days
# Treatment group
B5 <- pop_pchaz(T = times,
                   lambdaMat1    = m2r(matrix(c(11, 30,
                                                 11, 18), byrow = TRUE, nrow = 2)),
                   lambdaMat2    = m2r(matrix(c( 9, 20,
                                                  9, 11), byrow = TRUE, nrow = 2)),
                   lambdaProgMat = m2r(matrix(c( 5, 15,
                                                  5,  9), byrow = TRUE, nrow = 2)),
                   p = c(0.2, 0.8),#Proportion of subgroups
                discrete_approximation = TRUE
)
# Control group
K5  <- pop_pchaz(T = times,
                    lambdaMat1    = m2r(matrix(c(11, 11), nrow = 1)),
                    lambdaMat2    = m2r(matrix(c( 9,  9), nrow = 1)),
                    lambdaProgMat = m2r(matrix(c( 5,  5), nrow = 1)),
                    p = 1, discrete_approximation = TRUE
)
```

Then, using the resulting objects, we use them to generate a dataset with the `sample_fun` function:

```
# Study set up and Simulation of a data set until interim analysis at 150 events
set.seed(15657)
dat <- sample_fun(K5, B5,
                  r0 = 0.5,                      # Allocation ratio
                  eventEnd = 450,                # maximal number of events
                  lambdaRecr = 300 / 365,        # recruitment rate per day (Poisson assumption)
                  lambdaCens = 0.013 / 365,      # censoring rate per day  (Exponential assumption)
                  maxRecrCalendarTime = 3 * 365,# Maximal duration of recruitment
                  maxCalendar = 4 * 365.25)      # Maximal study duration
head(dat)
#>     group inclusion  y yCalendar event adminCens cumEvents
#> 777     1        26  3        29  TRUE     FALSE         1
#> 17      1        25 18        43  TRUE     FALSE         2
#> 367     1         9 40        49  TRUE     FALSE         3
#> 64      0        42  9        51  TRUE     FALSE         4
#> 25      0        34 40        74  TRUE     FALSE         5
#> 708     1       103  7       110  TRUE     FALSE         6
tail(dat)
#>     group inclusion   y yCalendar event adminCens cumEvents
#> 889     1       755 207       962 FALSE      TRUE       450
#> 893     0       205 757       962 FALSE      TRUE       450
#> 896     0       814 148       962 FALSE      TRUE       450
#> 900     1       510 452       962 FALSE      TRUE       450
#> 907     1       225 737       962 FALSE      TRUE       450
#> 908     0       717 245       962 FALSE      TRUE       450
```

The weighted log-rank test and the max-LRtest

The weighted log-rank test is implemented using the function `logrank.test`, which uses the statistic:

$$z = \sum_{t \in \mathcal{D}} w(t)(d_{t,ctr} - e_{t,ctr}) / \sqrt{\sum_{t \in \mathcal{D}} w(t)^2 var(d_{t,ctr})}.$$

where $w(t)$ are the Fleming-Harrington $\rho - \gamma$ family weights, such that $w(t) = \widehat{S}(t)^\rho (1 - \widehat{S}(t))^\gamma$. Under the the least favorable configuration in H_0, the test statistic is asymptotically standard normally distributed and large values of z are in favor of the alternative.

For example, the following code performs the weighted log-rank test using the simulated dataset and $\rho = 1$ and $\gamma = 0$.

```
logrank.test(time  = dat$y,
             event = dat$event,
             group = dat$group,
             # alternative = "greater",
             rho   = 1,
             gamma = 0)
#> Call:
#> logrank.test(time = dat$y, event = dat$event, group = dat$group,
#>     rho = 1, gamma = 0)
#>
#>     N Observed Expected (O-E)^2/E (O-E)^2/V
#> 1 411      267      211      15.2      58.3
#> 2 385      183      239      13.3      58.3
#>
#>  Chisq= 22  on 1 degrees of freedom, p= 3e-06
#>  rho   =  1 gamma =  0
# survival::survdiff(formula = survival::Surv(time  = dat$y, event = dat$event) ~ dat$group)
```

For a set of k different weight functions $w_1(t), \ldots, w_k(t)$, the maximum log-rank test statistic is $z_{max} = \max_{i=1,\ldots,k} z_i$. Under the least favorable configuration in H_0, approximately $(Z_1, \ldots, Z_k) \sim N_k(0, \Sigma)$. The p-value of the maximum test, $P_{H_0}(Z_{max} > z_{max})$, is calculated based on this multivariate normal approximation via numeric integration.

The following code performs the maximum log-rank test using four combinations of ρ and γ for the weights.

```
lrmt = logrank.maxtest(
      time  = dat$y,
      event = dat$event,
      group = dat$group,
      rho   = c(0, 0, 1, 1),
      gamma = c(0, 1, 0, 1)
)
lrmt
#> Call:
#> logrank.maxtest(time = dat$y, event = dat$event, group = dat$group,
#>     rho = c(0, 0, 1, 1), gamma = c(0, 1, 0, 1))
#>
#>  Two sided p-value = 4.91e-08 (Bonferroni corrected: 1.96e-07)
#>
```

```
#>  Individual weighted log-rank tests:
#>   Test    z        p
#> 1    1 5.37 7.99e-08
#> 2    2 5.24 1.58e-07
#> 3    3 4.69 2.76e-06
#> 4    4 5.45 4.91e-08
```

The individual tests can also be accesed using the `testListe` elements in the resulting object.

```
lrmt$logrank.test[[1]]
#> Call:
#> logrank.test(time = time, event = event, group = group, alternative = alternative,
#>     rho = rho[i], gamma = gamma[i])
#>
#>     N Observed Expected (O-E)^2/E (O-E)^2/V
#> 1 411      267      211      15.2      28.8
#> 2 385      183      239      13.3      28.8
#>
#>  Chisq= 28.8  on 1 degrees of freedom, p= 8e-08
#>  rho   =  0 gamma =  0
lrmt$logrank.test[[2]]
#> Call:
#> logrank.test(time = time, event = event, group = group, alternative = alternative,
#>     rho = rho[i], gamma = gamma[i])
#>
#>     N Observed Expected (O-E)^2/E (O-E)^2/V
#> 1 411      267      211      15.2       185
#> 2 385      183      239      13.3       185
#>
#>  Chisq= 27.5  on 1 degrees of freedom, p= 2e-07
#>  rho   =  0 gamma =  1
lrmt$logrank.test[[3]]
#> Call:
#> logrank.test(time = time, event = event, group = group, alternative = alternative,
#>     rho = rho[i], gamma = gamma[i])
#>
#>     N Observed Expected (O-E)^2/E (O-E)^2/V
#> 1 411      267      211      15.2      58.3
#> 2 385      183      239      13.3      58.3
#>
#>  Chisq= 22  on 1 degrees of freedom, p= 3e-06
#>  rho   =  1 gamma =  0
lrmt$logrank.test[[4]]
#> Call:
#> logrank.test(time = time, event = event, group = group, alternative = alternative,
#>     rho = rho[i], gamma = gamma[i])
#>
#>     N Observed Expected (O-E)^2/E (O-E)^2/V
#> 1 411      267      211      15.2       806
#> 2 385      183      239      13.3       806
#>
#>  Chisq= 29.8  on 1 degrees of freedom, p= 5e-08
#>  rho   =  1 gamma =  1
```

Examples

Consider a clinical trial in which patients are randomised to either an experimental treatment or a control one. The considered endpoint is survival time and the follow-up time is 5-years.

Progression of disease

The first example we consider is non-proportional hazards due to the case when there is progression of disease that affects the survival of the patients. Moreover, since the progression appears with different rates for treatment and control arms, it leads to a violation of the non-proportional assumption.

As an example, consider the case where the median time to progression of disease is 9 months for the experimental treatment (TRT) and 5 months for the control one (CTRL). Before progression, the median overall survival (OS) times are 18 (TRT) and 9 (CTRL) and after progression they decrease to 11 (TRT) and 9 (CTRL).

This is example is roughly based on the results observed in the study Keynote 189 (NEJM issue:22; 2018)

```
times <- c(0, 5 * 365)   # Time interval boundaries, in days

# Treatment group
t_resp <- 1              # There are no subgroups
B5 <- pop_pchaz(
  T = times,
  lambdaMat1     = m2r(matrix(18,nrow = 1)),
  lambdaMat2     = m2r(matrix(11,nrow = 1)),
  lambdaProgMat = m2r(matrix(9, nrow = 1)),
  p = t_resp,
  timezero = FALSE, discrete_approximation = TRUE
)

# Control group
c_resp <- 1              # There are no subgroups
K5  <- pop_pchaz(
  T = times,
  lambdaMat1     = m2r(matrix(11,nrow = 1)),
  lambdaMat2     = m2r(matrix(9, nrow = 1)),
  lambdaProgMat = m2r(matrix(5, nrow = 1)),
  p = c_resp,
  timezero = TRUE, discrete_approximation = TRUE
)

pp = plot_diagram(B5, K5)
pp
```

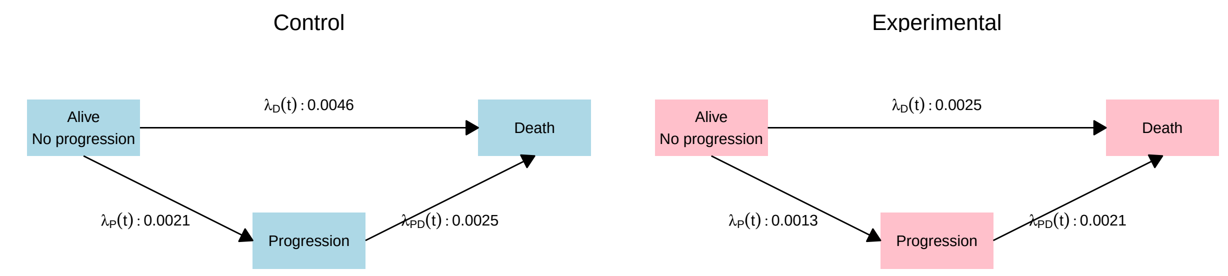

The plot shows us the the survival and hazard functions by group, together with the hazard ratio. We observe that the HR is not constant through time and conclude that progression of disease leads to a non-proportional hazard.

```
plot_shhr(K5, B5)
```

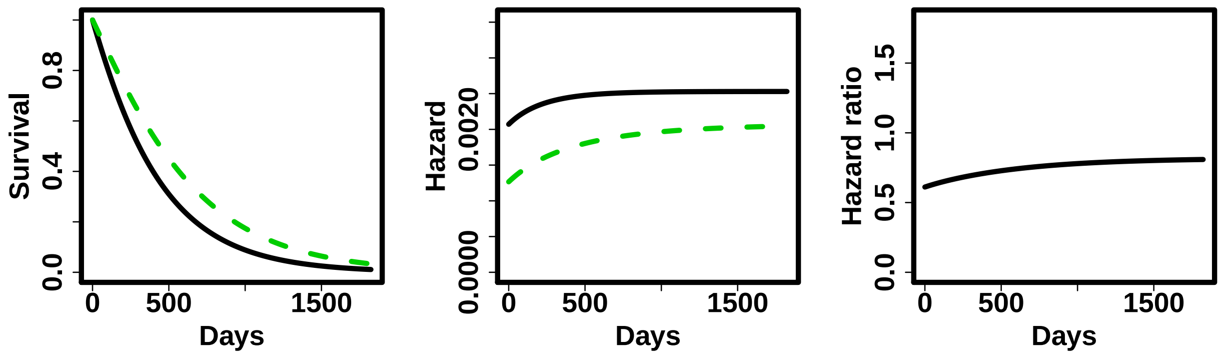

Subgroup with differential effect

Another feature that may lead a violation of the non-proportional hazards assumption is the presence of predictive subgroup. That is, subgroups with a differential treatment effect. We then build on the previous example to include a subgroup (with prevalence of 20%) with an additional benefit. The median time to progression in the subgroup is 15mo, while the median OS is 30mo before progression and 20mo after progression. For the complement of the subgroup, the parameters are maintained at the values of the previous example.

```
times <- c(0, 5 * 365)    # Time interval boundaries, in days

# Treatment group
t_resp <-  c(.2, .8)
B5 <- pop_pchaz(
  T = times,
  lambdaMat1     = m2r(matrix(c(30,
```

```
                               18),nrow = 2)),
  lambdaMat2    = m2r(matrix(c(20,
                               11),nrow = 2)),
  lambdaProgMat = m2r(matrix(c(15,
                               9), nrow = 2)),
  p = t_resp,
  timezero = FALSE, discrete_approximation = TRUE
)

# Control group
c_resp <- 1
K5  <- pop_pchaz(
  T = times,
  lambdaMat1    = m2r(matrix(11,nrow = 1)),
  lambdaMat2    = m2r(matrix(9, nrow = 1)),
  lambdaProgMat = m2r(matrix(5, nrow = 1)),
  p = c_resp,
  timezero = TRUE, discrete_approximation = TRUE
)

pp = plot_diagram(B5, K5, A_subgr_labels = c("S1", "S2"))
pp
```

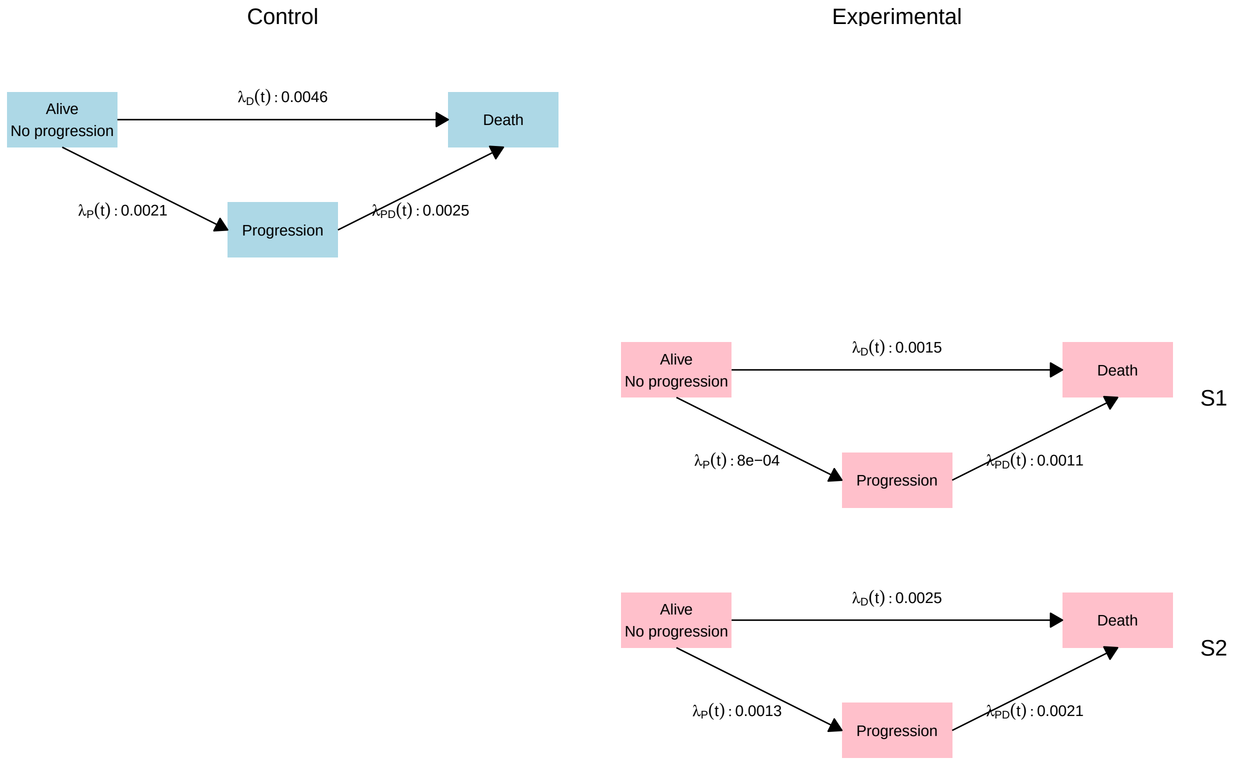


```r
plot_shhr(K5, B5)
```

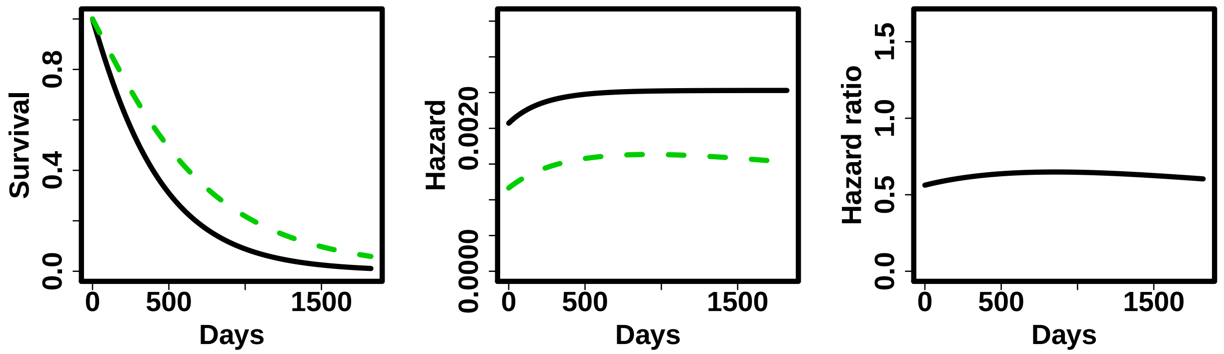

Note that the hazard ratio now does not monotonically increase. Curiously, when we assume a larger prevalence of the subgroup, the HR is more flat across time and the violation of the non-proportionality is atenuated. As two different mechamisms, progression and predictive subgroup, are affecting the pattern of

the HR, it may not be straightforward to know in advance whether the non-proportional hazards assumption will be met or not. The **nph** package is of great help to model and visualise possible patterns.

```
times <- c(0, 5 * 365)    # Time interval boundaries, in days

# Treatment group
t_resp <-  c(.5, .5)
B5 <- pop_pchaz(
  T = times,
  lambdaMat1    = m2r(matrix(c(30,
                               18),nrow = 2)),
  lambdaMat2    = m2r(matrix(c(20,
                               11),nrow = 2)),
  lambdaProgMat = m2r(matrix(c(15,
                               9), nrow = 2)),
  p = t_resp,
  timezero = FALSE, discrete_approximation = TRUE
)

# Control group
c_resp <- 1
K5  <- pop_pchaz(
  T = times,
  lambdaMat1    = m2r(matrix(11,nrow = 1)),
  lambdaMat2    = m2r(matrix(9, nrow = 1)),
  lambdaProgMat = m2r(matrix(5, nrow = 1)),
  p = c_resp,
  timezero = TRUE, discrete_approximation = TRUE
)
```

```
plot_shhr(K5, B5)
```

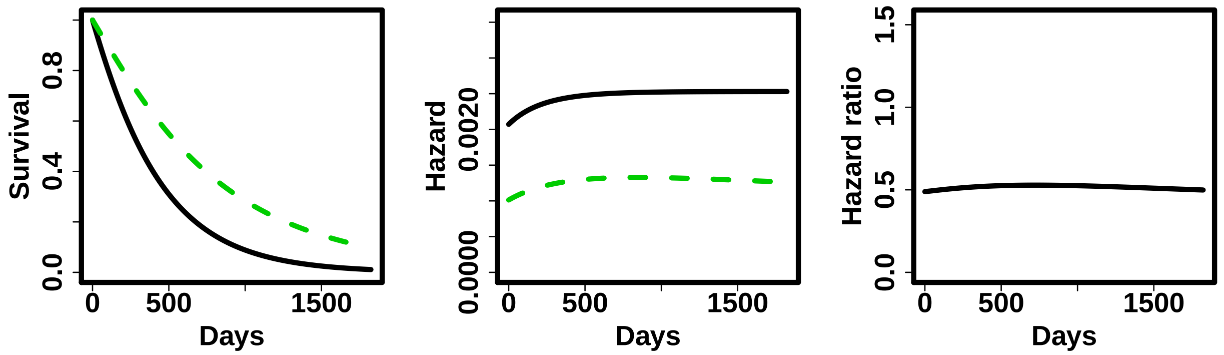

Further assume that the median OS is the same before and after progression for the subgroup. This is usually reffered to as the "cure" model.

```
times <- c(0, 5 * 365)    # Time interval boundaries, in days

# Treatment group
t_resp <-  c(.5, .5)
```

```r
B5 <- pop_pchaz(
  T = times,
  lambdaMat1    = m2r(matrix(c(30,
                               18), nrow = 2)),
  lambdaMat2    = m2r(matrix(c(30,
                               11), nrow = 2)),
  lambdaProgMat = m2r(matrix(c(15,
                                9), nrow = 2)),
  p = t_resp,
  timezero = FALSE, discrete_approximation = TRUE
)

# Control group
c_resp <- 1
K5  <- pop_pchaz(
  T = times,
  lambdaMat1    = m2r(matrix(11, nrow = 1)),
  lambdaMat2    = m2r(matrix( 9, nrow = 1)),
  lambdaProgMat = m2r(matrix( 5, nrow = 1)),
  p = c_resp,
  timezero = TRUE, discrete_approximation = TRUE
)


pp = plot_diagram(B5, K5, A_subgr_labels = c("S1", "S2"))
pp
```

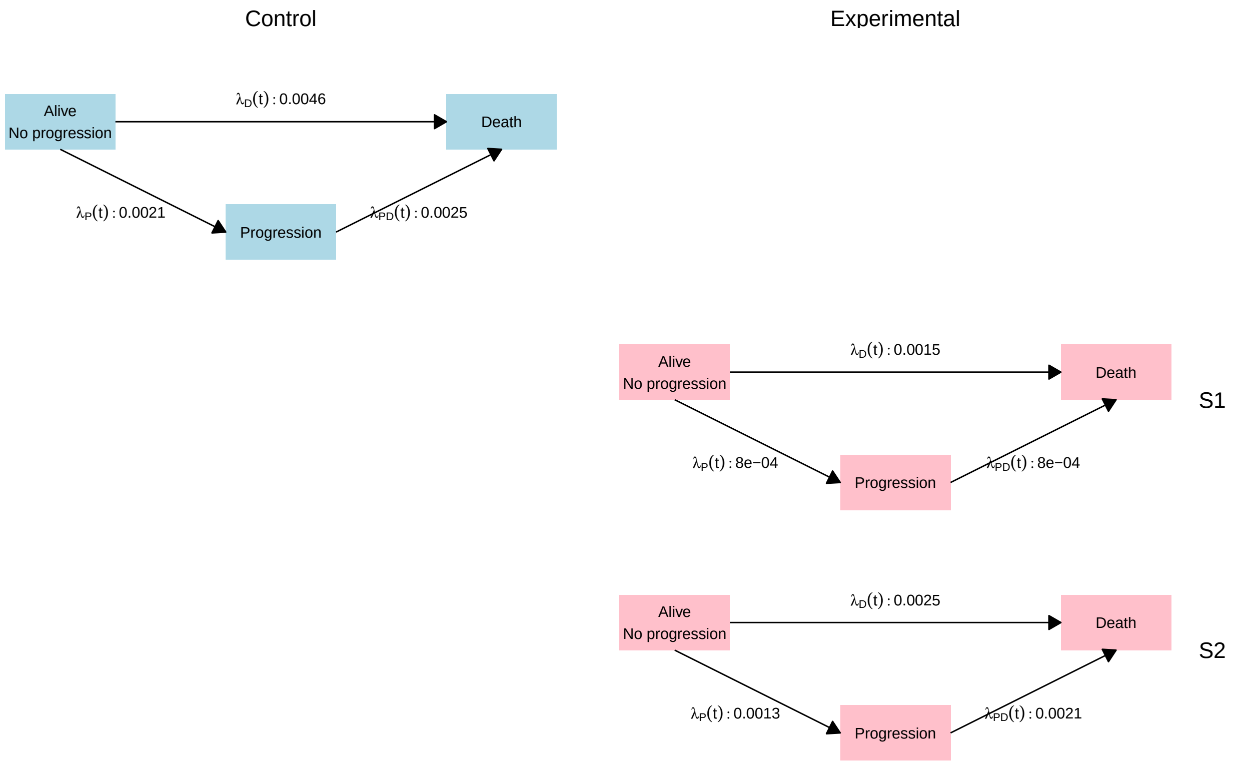


```
plot_shhr(K5, B5)
```

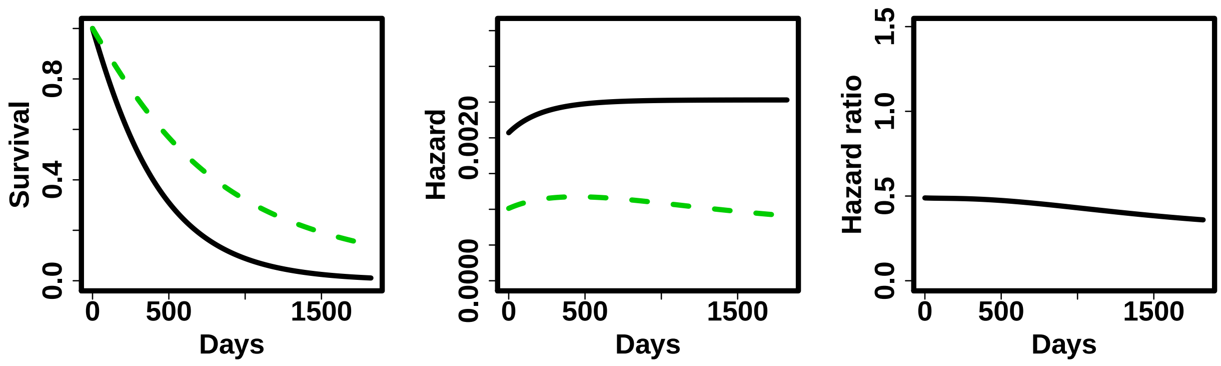

Delayed Effect

Another mechamisnm that affects the hazard ratio across time is a delayed drug effect. For instance, assume that in the first 100 days of treatment, the experimental and control treatments have the same median survival time. After day 100 ($t > 100$), the experimental treatment has a longer OS (median OS 18 and 11 before and after progression, respectively).

```
times <- c(0, 100, 5 * 365)    # Time interval boundaries, in days

# Treatment group
t_resp <- 1                 # There are no subgroups
B5 <- pop_pchaz(
  T = times,
  lambdaMat1     = m2r(matrix(c(11, 18), nrow = 1)),
  lambdaMat2     = m2r(matrix(c( 9, 11), nrow = 1)),
  lambdaProgMat = m2r(matrix(c( 5,  9), nrow = 1)),
  p = t_resp,
  timezero = FALSE, discrete_approximation = TRUE
)

# Control group
c_resp <- 1
K5  <- pop_pchaz(
  T = times,
  lambdaMat1     = m2r(matrix(c(11, 11), nrow = 1)),
  lambdaMat2     = m2r(matrix(c( 9,  9), nrow = 1)),
  lambdaProgMat = m2r(matrix(c( 5,  5), nrow = 1)),
  p = c_resp,
  timezero = TRUE, discrete_approximation = TRUE
)


pp = plot_diagram(B5, K5)
pp
```

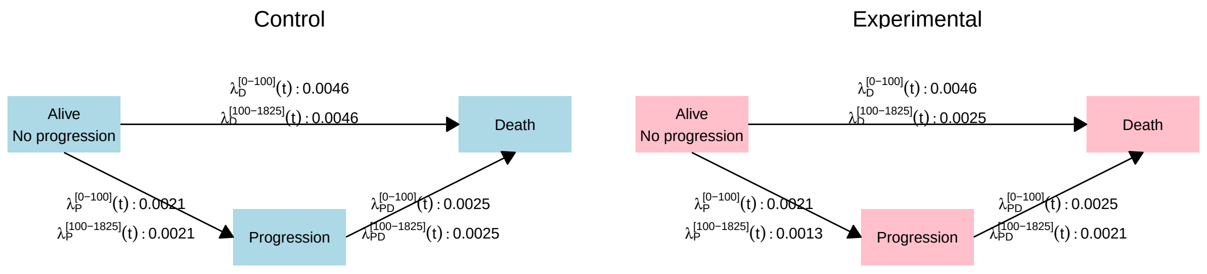


```
plot_shhr(K5, B5)
```

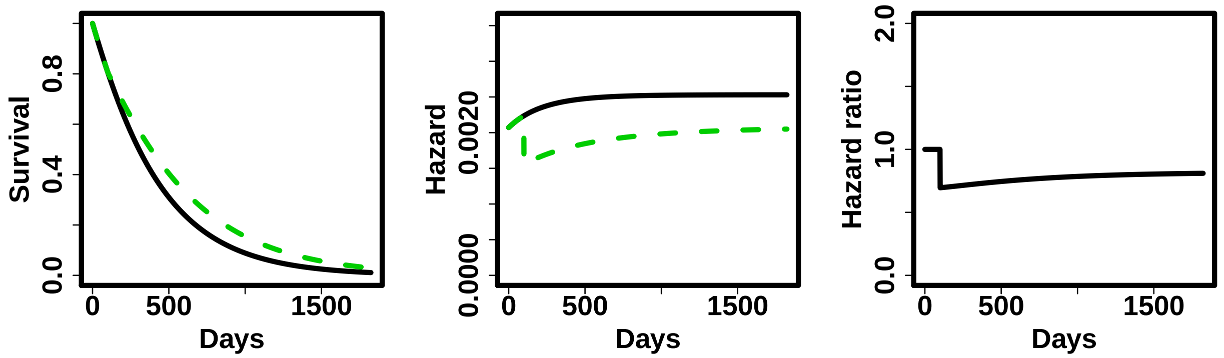

Delayed Effect and Subgroup with differential effect

It is also possible to combine a delayed effect and a predictive subgroup. In this case, from days 0 to 100, the median survival times are the same for the control and experimental treatments and in both subgroups. As before, the drug starts providing a benefit after 100 days, but the subgroup has an additional benefit.

```
times <- c(0, 100, 5 * 365)   # Time interval boundaries, in days

# Treatment group
t_resp <- c(0.5, 0.5) #Proportion of subgroups
B5 <- pop_pchaz(
  T = times,
    lambdaMat1    = m2r(matrix(c(11, 30,
                                 11, 18), byrow = TRUE, nrow = 2)),
    lambdaMat2    = m2r(matrix(c( 9, 20,
                                  9, 11), byrow = TRUE, nrow = 2)),
    lambdaProgMat = m2r(matrix(c( 5, 15,
                                  5,  9), byrow = TRUE, nrow = 2)),
  p = t_resp,
  timezero = FALSE, discrete_approximation = TRUE
)

# Control group
c_resp <- 1
K5  <- pop_pchaz(
  T = times,
  lambdaMat1    = m2r(matrix(c(11, 11), nrow = 1, ncol = 2)),
  lambdaMat2    = m2r(matrix(c( 9,  9), nrow = 1, ncol = 2)),
  lambdaProgMat = m2r(matrix(c( 5,  5), nrow = 1, ncol = 2)),
  p = c_resp,
  timezero = TRUE, discrete_approximation = TRUE
)

pp = plot_diagram(B5, K5, A_subgr_labels = c("S1", "S2"))
pp
```

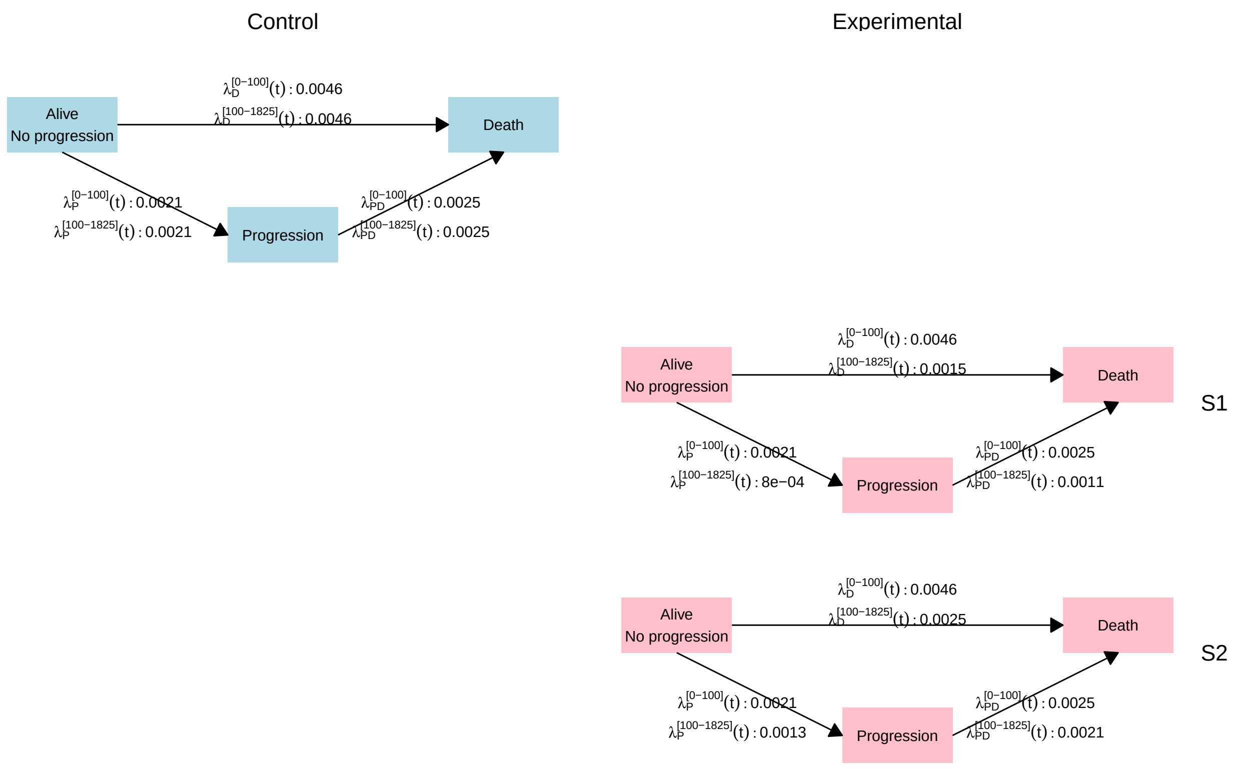


```r
plot_shhr(K5, B5)
```

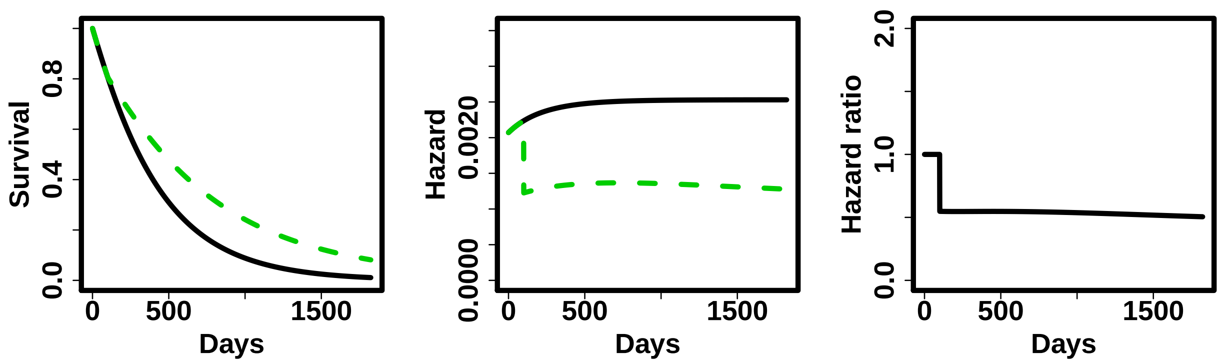

Treatment Switchers

The last mechanism we explore for non-proportionality is when there are treatment switchers. In some clinical trials, patients under the control treatment are provided with the experimental treatment after their disease progresses. However, in the analysis phase, these patients will still be considered in the control group according to the intention-to-treat principle.

Assume then that after progression, 2/3 of the patients in the control treatment switch to the experimental one and therefore they have the same median survival time as in the treatment group after progression.

```
times <- c(0, 5 * 365)   # Time interval boundaries, in days

# Treatment group
t_resp <- 1               # There are no subgroups
B5 <- pop_pchaz(
  T = times,
  lambdaMat1    = m2r(matrix(18, nrow = 1)),
  lambdaMat2    = m2r(matrix(11, nrow = 1)),
  lambdaProgMat = m2r(matrix( 9, nrow = 1)),
  p = t_resp,
  timezero = FALSE, discrete_approximation = TRUE
)

# Control group
c_resp <- c(1/3, 2/3) #non-switcher, switcher reponse
K5  <- pop_pchaz(
  T = times,
  lambdaMat1    = m2r(matrix(c(11,
                               11), nrow = 2, ncol = 1)),
  lambdaMat2    = m2r(matrix(c( 9,
                               11), nrow = 2, ncol = 1)),
  lambdaProgMat = m2r(matrix(c( 5,
                                5), nrow = 2, ncol = 1)),
  p = c_resp,
  timezero = TRUE, discrete_approximation = TRUE
)


pp = plot_diagram(B5, K5, B_subgr_labels = c("Non-switcher","Switcher"))

pp
```

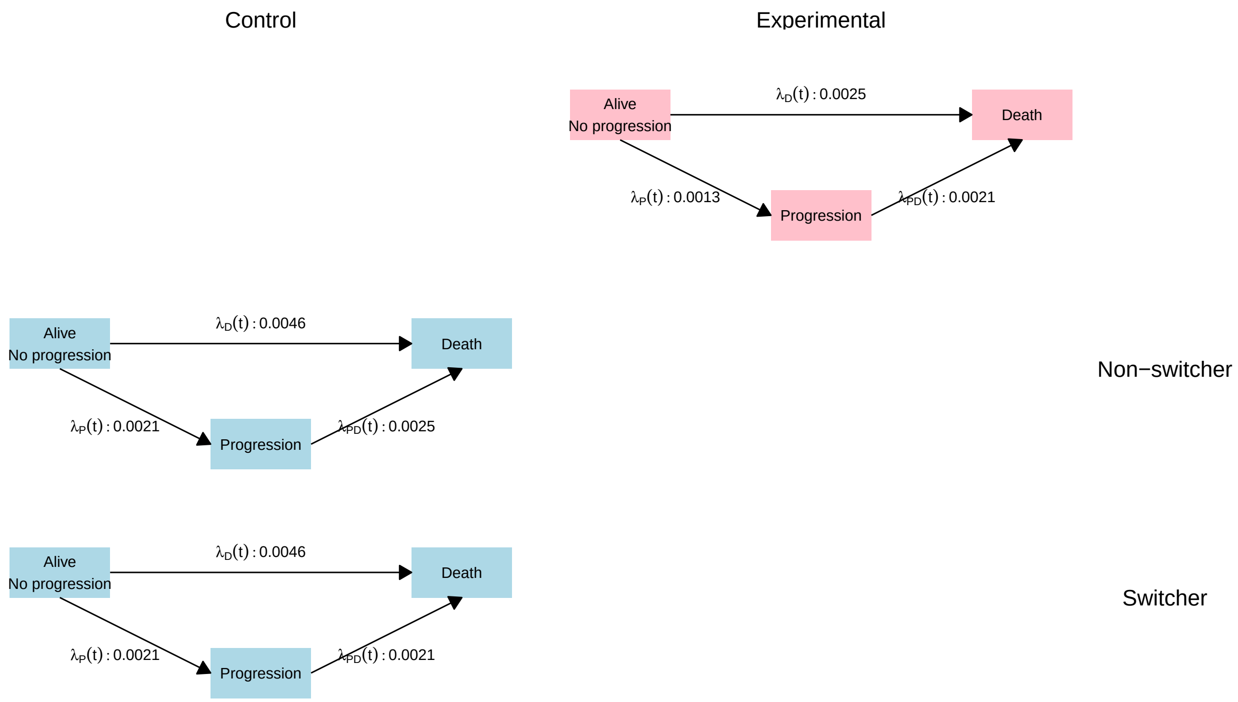


```r
plot_shhr(K5, B5)
```

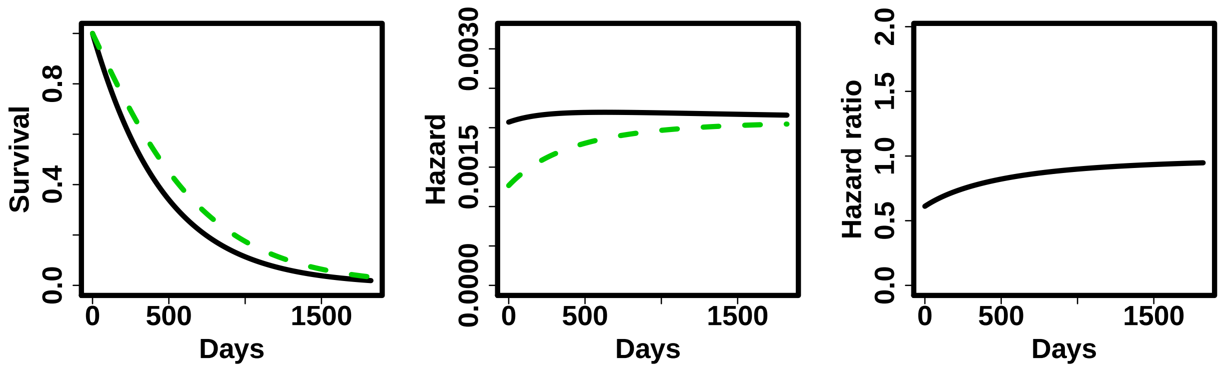

It would also be sensible to assume that switchers have a higher median survival, but slightly lower median survival time when compared to the treatment group before progression.

```r
times <- c(0, 5 * 365)   # Time interval boundaries, in days

# Treatment group
t_resp <- 1                # There are no subgroups
B5 <- pop_pchaz(
  T = times,
  lambdaMat1    = m2r(matrix(18, nrow = 1)),
  lambdaMat2    = m2r(matrix(11, nrow = 1)),
  lambdaProgMat = m2r(matrix( 9, nrow = 1)),
  p = t_resp,
  timezero = FALSE, discrete_approximation = TRUE
)

# Control group
c_resp <- c(1/3, 2/3) #non-switcher, switcher reponse
K5  <- pop_pchaz(
  T = times,
  lambdaMat1    = m2r(matrix(c(11,
                               11), nrow = 2, ncol = 1)),
  lambdaMat2    = m2r(matrix(c( 9,
                               14), nrow = 2, ncol = 1)),
  lambdaProgMat = m2r(matrix(c( 5,
                                5), nrow = 2, ncol = 1)),
  p = c_resp,
  timezero = TRUE, discrete_approximation = TRUE
)

pp = plot_diagram(B5, K5, B_subgr_labels = c("Non-switcher","Switcher"))
pp
```

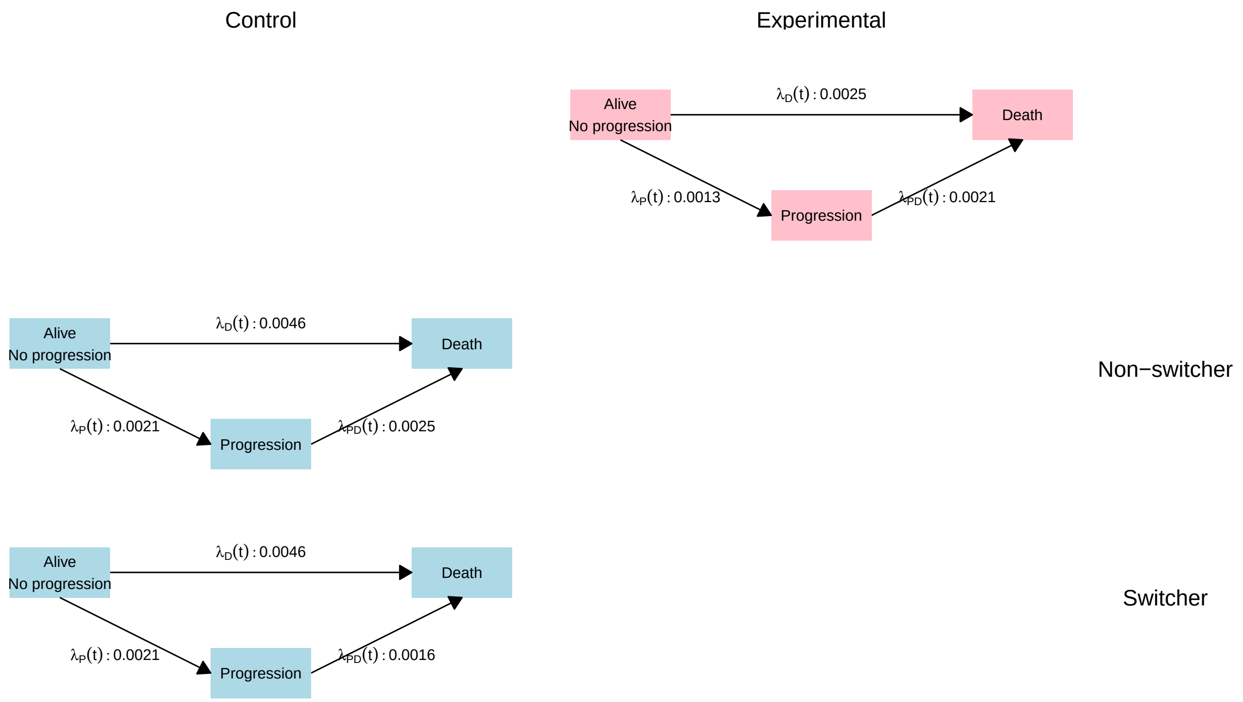


```r
plot_shhr(K5, B5)
```

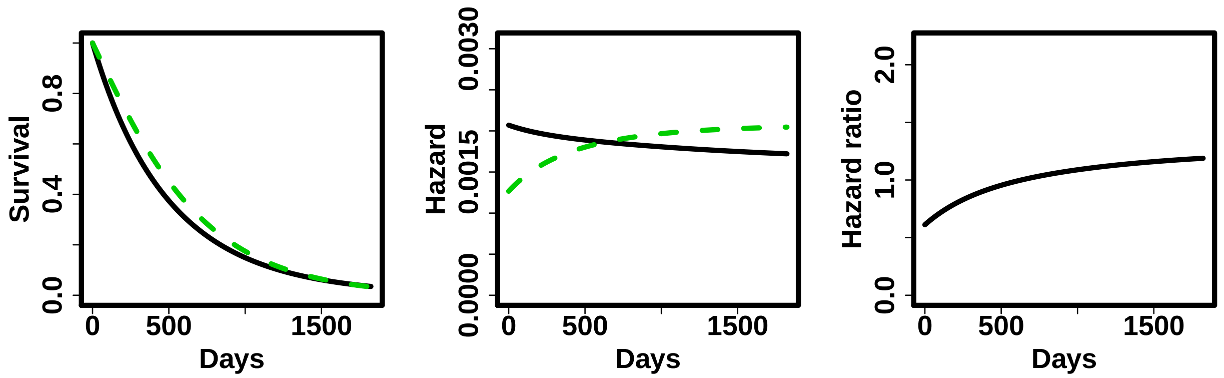

Different proportion of treatment switchers may have a different effect on the hazard ratio. For example, the following two cases are identical to the two previous ones, but we change the proportion of treatment switcher to 1/3 instead of 2/3.

```r
times <- c(0, 5 * 365)   # Time interval boundaries, in days

# Treatment group
t_resp <- 1                # There are no subgroups
B5 <- pop_pchaz(
  T = times,
  lambdaMat1    = m2r(matrix(18,nrow = 1)),
  lambdaMat2    = m2r(matrix(11,nrow = 1)),
  lambdaProgMat = m2r(matrix(9, nrow = 1)),
  p = t_resp,
  timezero = FALSE, discrete_approximation = TRUE
)

# Control group
c_resp <- c(2/3, 1/3) #non-switcher, switcher reponse
K5  <- pop_pchaz(
  T = times,
  lambdaMat1    = m2r(matrix(c(11,
                               11), nrow = 2, ncol = 1)),
  lambdaMat2    = m2r(matrix(c(9,
                               11), nrow = 2, ncol = 1)),
  lambdaProgMat = m2r(matrix(c(5,
                               5),  nrow = 2, ncol = 1)),
  p = c_resp,
  timezero = TRUE, discrete_approximation = TRUE
)

pp = plot_diagram(B5, K5, B_subgr_labels = c("Non-switcher","Switcher"))
pp
```

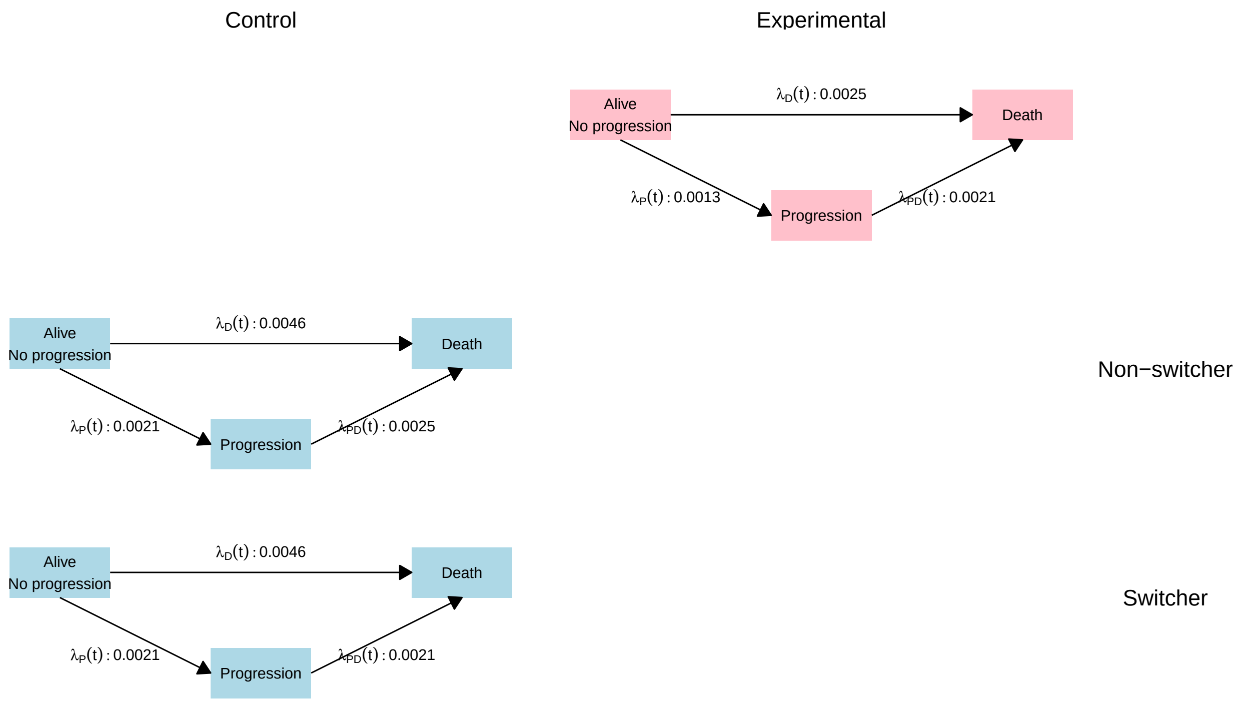


```r
plot_shhr(K5, B5)
```

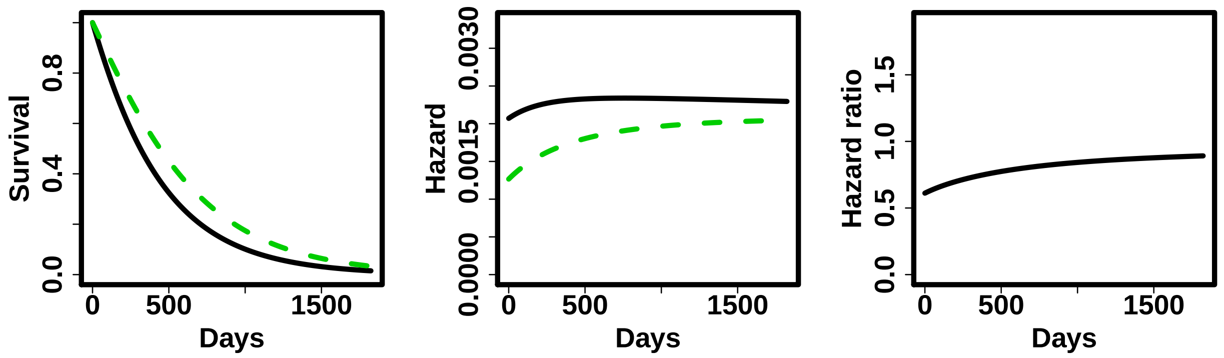


```r
times <- c(0, 5 * 365)    # Time interval boundaries, in days

# Treatment group
```

```r
t_resp <- 1                # There are no subgroups
B5 <- pop_pchaz(
  T = times,
  lambdaMat1    = m2r(matrix(18, nrow = 1)),
  lambdaMat2    = m2r(matrix(11, nrow = 1)),
  lambdaProgMat = m2r(matrix( 9, nrow = 1)),
  p = t_resp,
  timezero = FALSE, discrete_approximation = TRUE
)

# Control group
c_resp <- c(2/3, 1/3) #non-switcher, switcher reponse
K5  <- pop_pchaz(
  T = times,
  lambdaMat1    = m2r(matrix(c(11,
                               11), nrow = 2, ncol = 1)),
  lambdaMat2    = m2r(matrix(c( 9,
                               14), nrow = 2, ncol = 1)),
  lambdaProgMat = m2r(matrix(c( 5,
                                5), nrow = 2, ncol = 1)),
  p = c_resp,
  timezero = TRUE, discrete_approximation = TRUE
)

pp = plot_diagram(B5, K5, B_subgr_labels = c("Non-switcher","Switcher"))
pp
```

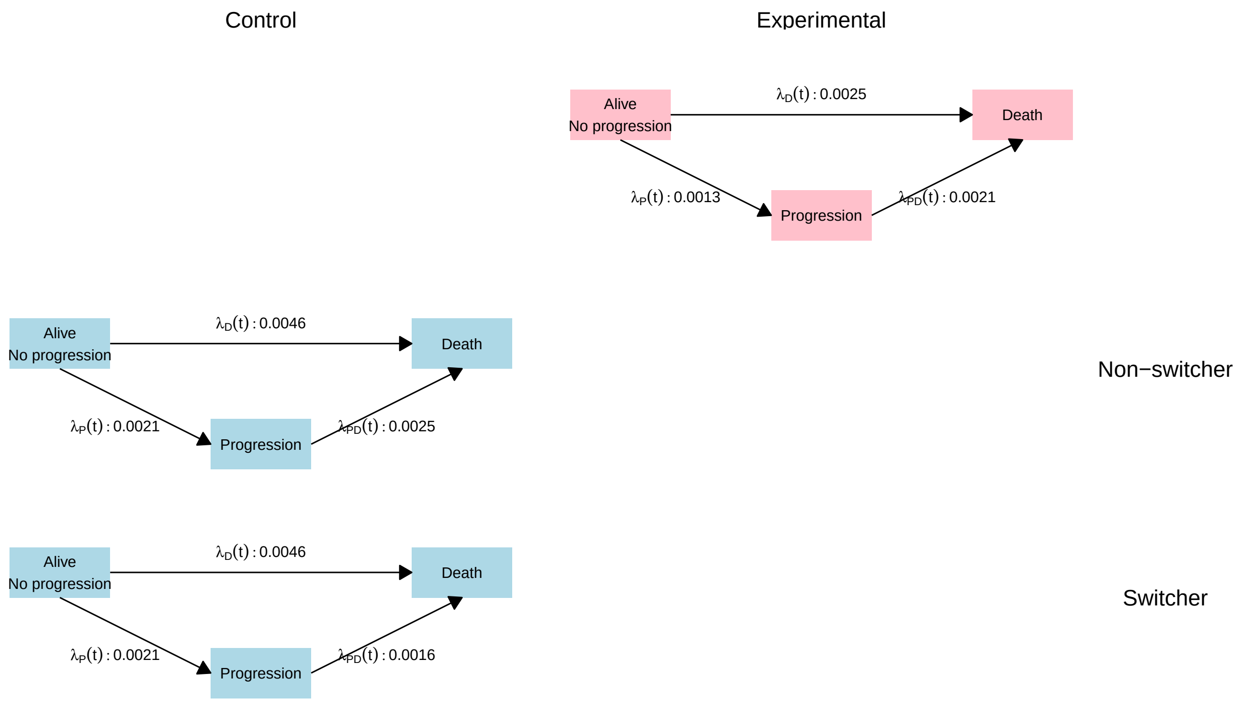


```r
plot_shhr(K5, B5)
```

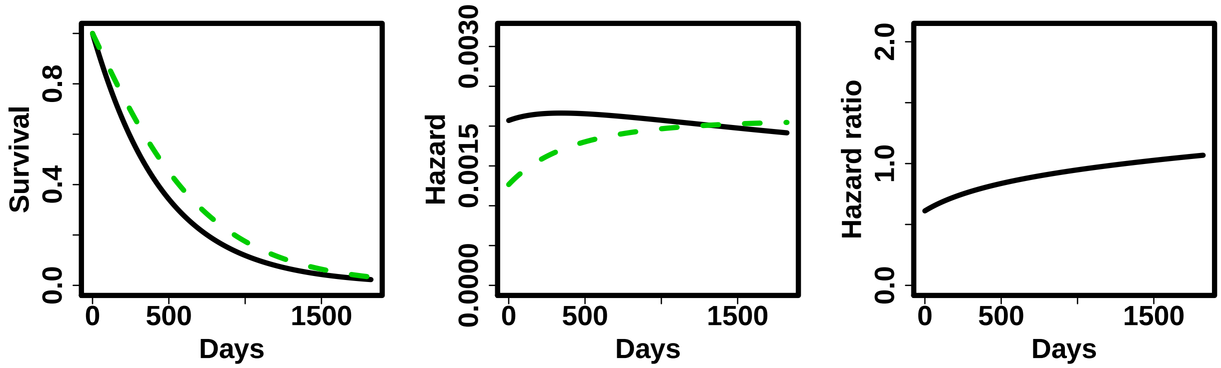

Harmful drug in a subgroup but beneficial in the complement.

Some interesting scenarios may arise. For example, consider of a drug that harms a subgroup decreasing their survival time, but providing benefit to the complement of the subgroup. This scenario results in crossing survival curves, which leads to a decreasing hazard ratio.

```r
times <- c(0, 5 * 365)   # Time interval boundaries, in days

# Treatment group
t_resp <- c(.5, .5)
B5 <- pop_pchaz(
  T = times,
    lambdaMat1    = m2r(matrix(c( 2,
                                 18), nrow = 2)),
    lambdaMat2    = m2r(matrix(c( 2,
                                 18), nrow = 2)),
    lambdaProgMat = m2r(matrix(c(19,
                                 19), nrow = 2)),
  p = t_resp,
  timezero = FALSE, discrete_approximation = TRUE
)

# Control group
c_resp <- c(1)
K5  <- pop_pchaz(
  T = times,
  lambdaMat1    = m2r(matrix(c(11), nrow = 1, ncol = 1)),
  lambdaMat2    = m2r(matrix(c( 9), nrow = 1, ncol = 1)),
  lambdaProgMat = m2r(matrix(c( 5), nrow = 1, ncol = 1)),
  p = c_resp,
  timezero = TRUE, discrete_approximation = TRUE
)

pp = plot_diagram(B5, K5,
                  A_subgr_labels = c("S1", "S2"))
pp
```

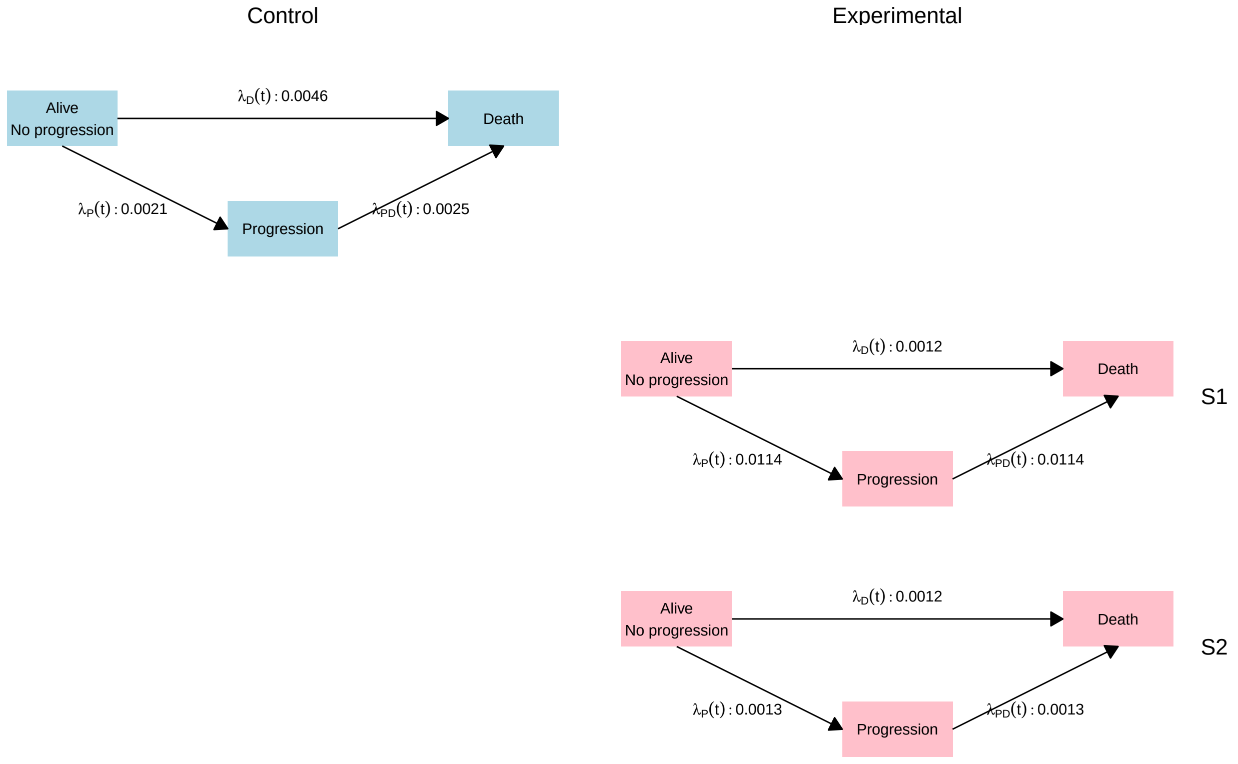


```
plot_shhr(K5, B5)
```

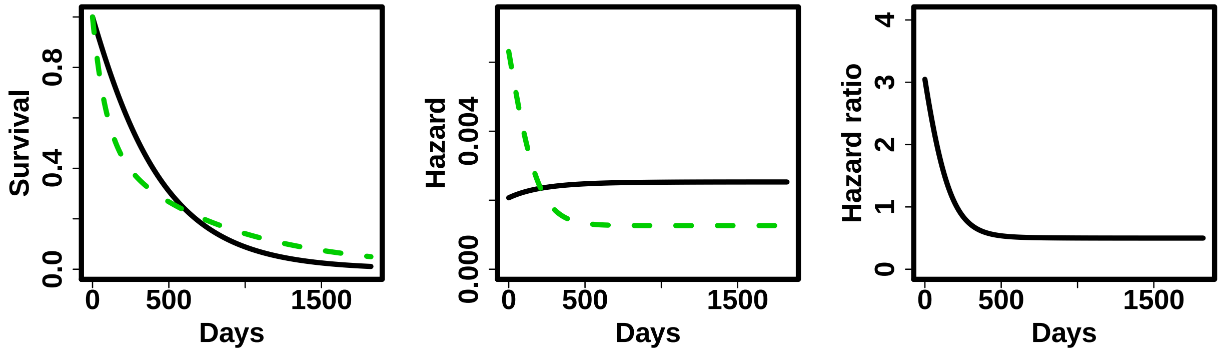

Switchers and subgroup with differential effect.

We now combine the case of treatment switchers with the case of a predictive subgroup. In this case, we also address the issue that the treatment switchers may also respond differently to the drug according to whether they belong to the predictive subgroup.

In the following cases we add the predictive subgroups with treatment switchers using different median survival and switchers proportions.

First, lets examine the cure model.

```
times <- c(0, 5 * 365)    # Time interval boundaries, in days

# Treatment group
t_resp <- c(.2, .8)
B5 <- pop_pchaz(
  T = times,
  lambdaMat1     = m2r(matrix(c(30,
                                18), nrow = 2)),
  lambdaMat2     = m2r(matrix(c(30,
                                11), nrow = 2)),
  lambdaProgMat = m2r(matrix(c(15,
                                 9), nrow = 2)),
  p = t_resp,
  timezero = FALSE, discrete_approximation = TRUE
)

# Control group
c_resp <- c(2/3, 1/3*t_resp[1], 1/3*t_resp[2])
K5  <- pop_pchaz(
  T = times,
  lambdaMat1     = m2r(matrix(c(11), nrow = 3, ncol = 1)),
  lambdaMat2     = m2r(matrix(c( 9,
                                25,
                                14), nrow = 3)),
  lambdaProgMat = m2r(matrix(c( 5), nrow = 3,ncol = 1)),
  p = c_resp,
  timezero = TRUE, discrete_approximation = TRUE
```

```
)

pp = plot_diagram(B5, K5,
                  A_subgr_labels = c("S1", "S2"),
                  B_subgr_labels = c("Non-switcher","Switcher-S1","Switcher-S2"))
pp
```

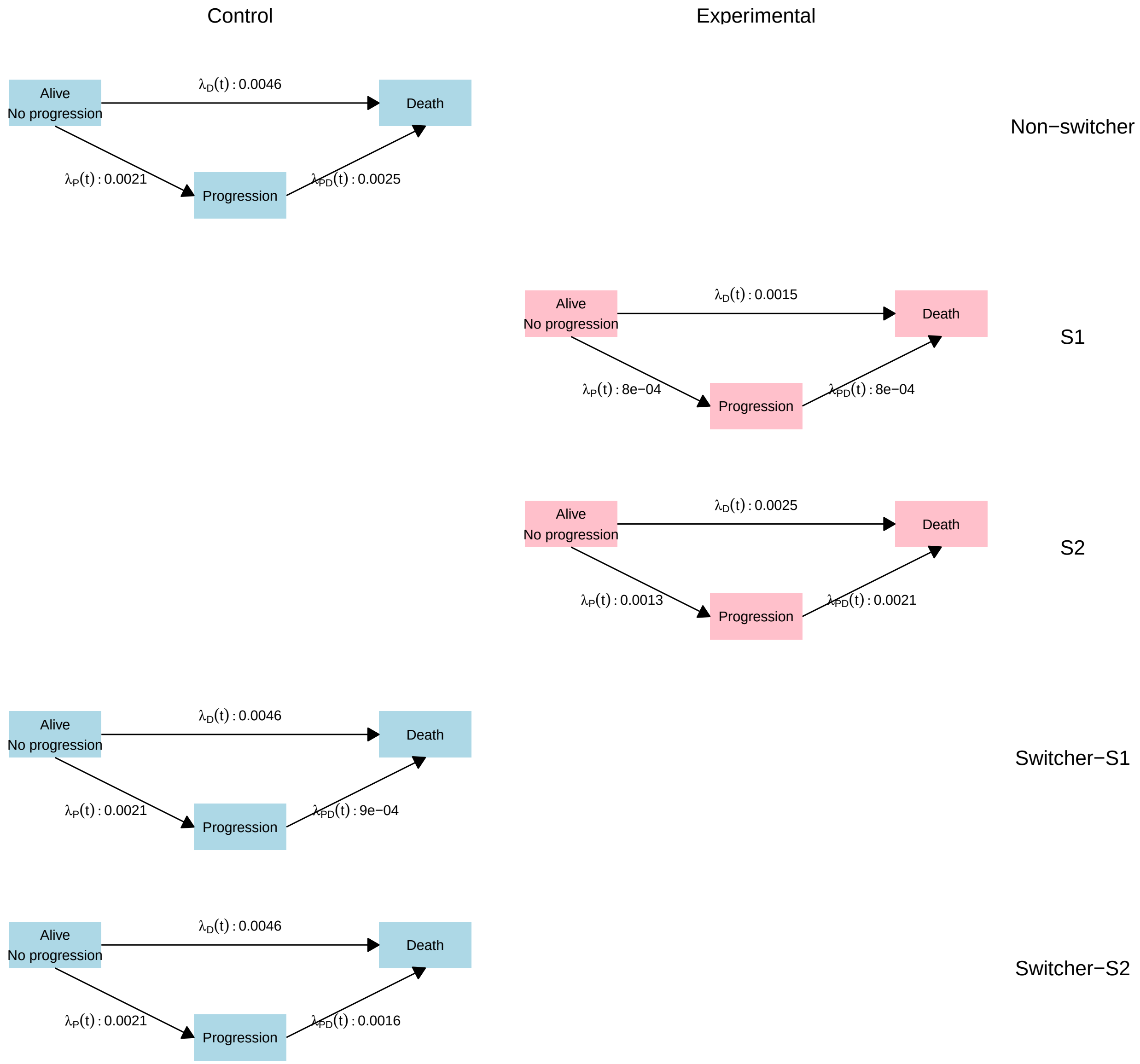


```
plot_shhr(K5, B5)
```

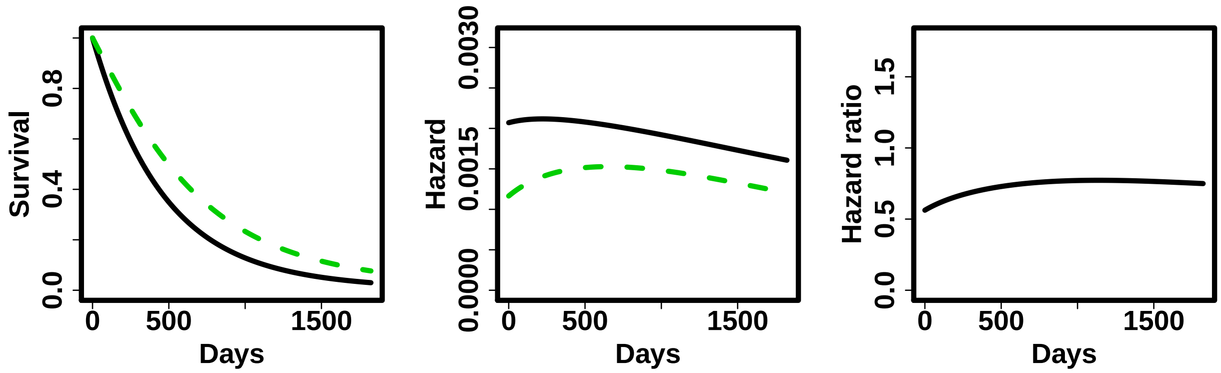

Now subgroup prevalence is 0.5.

```
times <- c(0, 5 * 365)   # Time interval boundaries, in days

# Treatment group
t_resp <- c(.5, .5)
B5 <- pop_pchaz(
  T = times,
  lambdaMat1    = m2r(matrix(c(30,
                               18), nrow = 2)),
  lambdaMat2    = m2r(matrix(c(30,
                               11), nrow = 2)),
  lambdaProgMat = m2r(matrix(c(15,
                                9), nrow = 2)),
  p = t_resp,
  timezero = FALSE, discrete_approximation = TRUE
)

# Control group
c_resp <- c(2/3, 1/3*t_resp[1], 1/3*t_resp[2])
K5  <- pop_pchaz(
  T = times,
  lambdaMat1    = m2r(matrix(c(11), nrow = 3, ncol = 1)),
  lambdaMat2    = m2r(matrix(c( 9,
                               25,
                               14), nrow = 3)),
  lambdaProgMat = m2r(matrix(c( 5), nrow = 3,ncol = 1)),
  p = c_resp,
  timezero = TRUE, discrete_approximation = TRUE
)

pp = plot_diagram(B5, K5,
                  A_subgr_labels = c("S1", "S2"),
                  B_subgr_labels = c("Non-switcher","Switcher-S1","Switcher-S2"))
pp
```

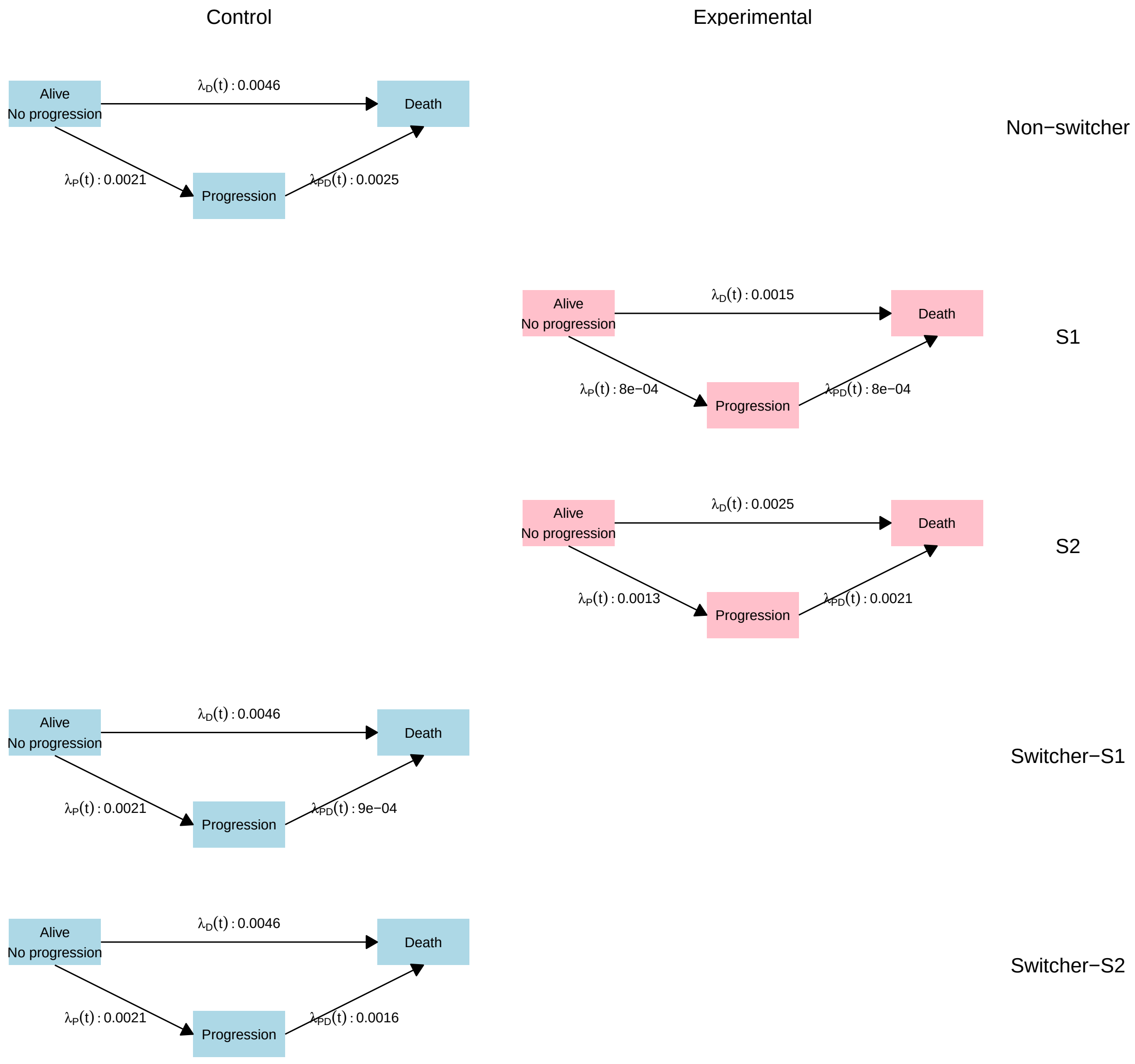


```
plot_shhr(K5, B5)
```

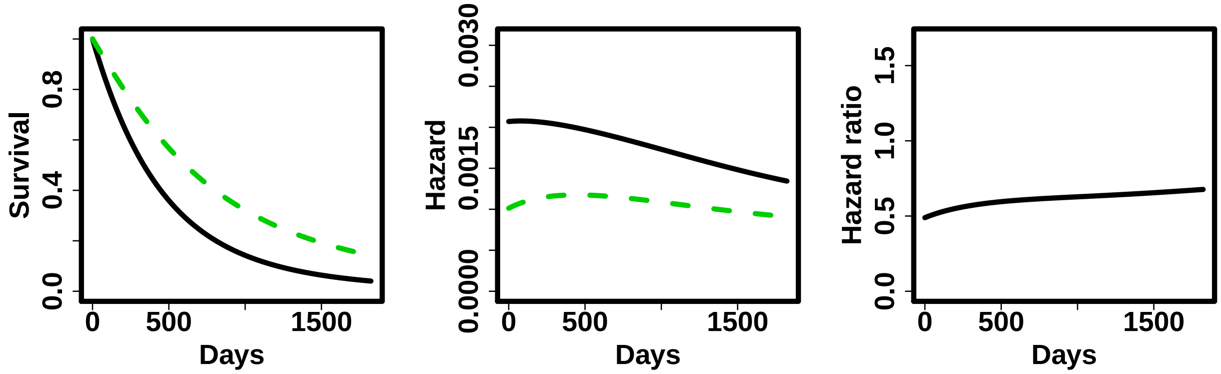

Now proportion of switchers is 2/3.

```
times <- c(0, 5 * 365)   # Time interval boundaries, in days

# Treatment group
t_resp <- c(.5, .5)
B5 <- pop_pchaz(
  T = times,
  lambdaMat1    = m2r(matrix(c(30,
                              18), nrow = 2)),
  lambdaMat2    = m2r(matrix(c(20,
                              11), nrow = 2)),
  lambdaProgMat = m2r(matrix(c(15,
                               9), nrow = 2)),
  p = t_resp,
  timezero = FALSE, discrete_approximation = TRUE
)

# Control group
c_resp <- c(1/3, 2/3*t_resp[1], 2/3*t_resp[2])
K5  <- pop_pchaz(
  T = times,
  lambdaMat1    = m2r(matrix(c(11), nrow = 3, ncol = 1)),
  lambdaMat2    = m2r(matrix(c( 9,
                              25,
                              14), nrow = 3)),
  lambdaProgMat = m2r(matrix(c( 5), nrow = 3,ncol = 1)),
  p = c_resp,
  timezero = TRUE, discrete_approximation = TRUE
)


pp = plot_diagram(B5, K5,
                  A_subgr_labels = c("S1", "S2"),
                  B_subgr_labels = c("Non-switcher","Switcher-S1","Switcher-S2"))
pp
```

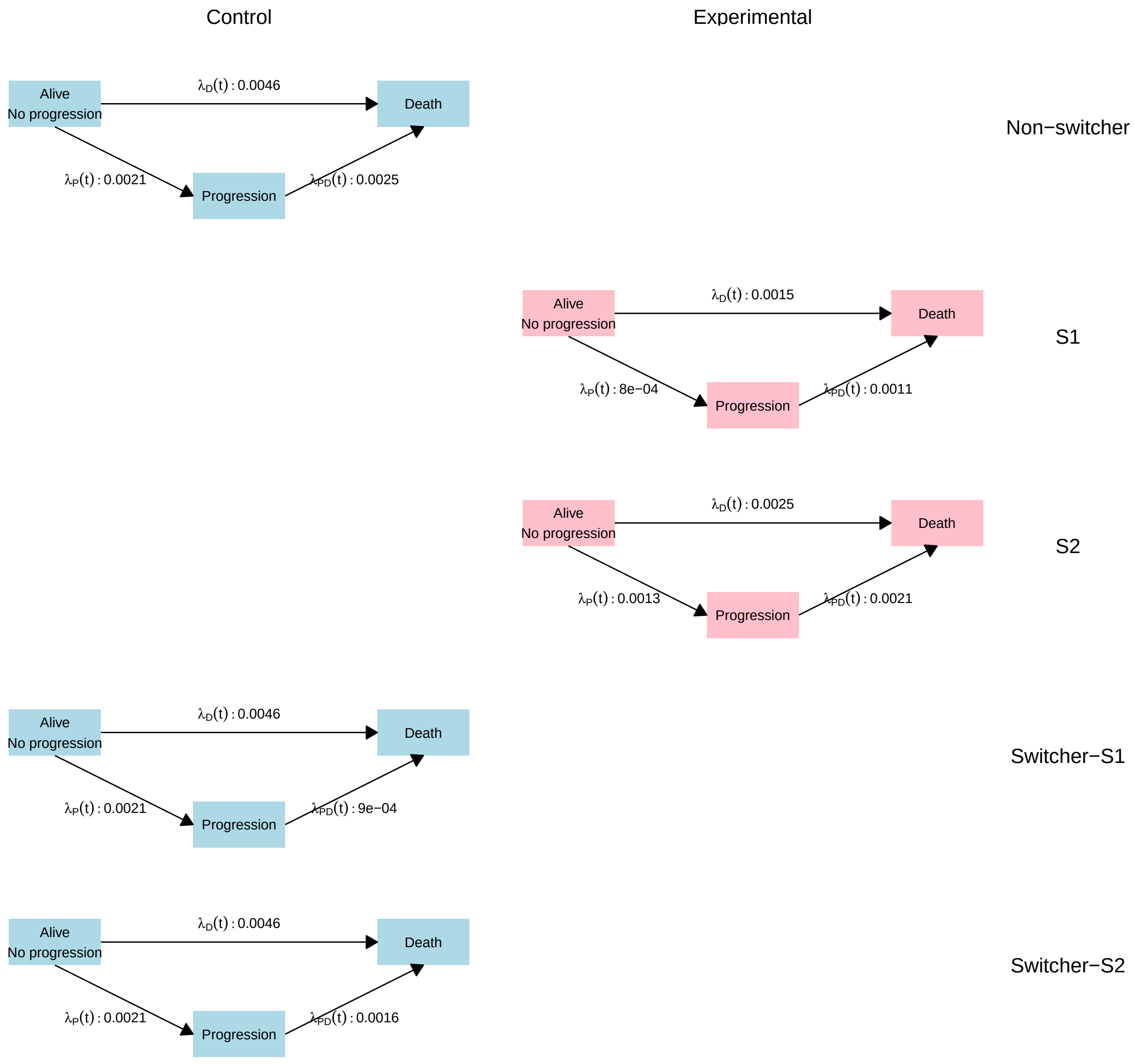


```
plot_shhr(K5, B5)
```

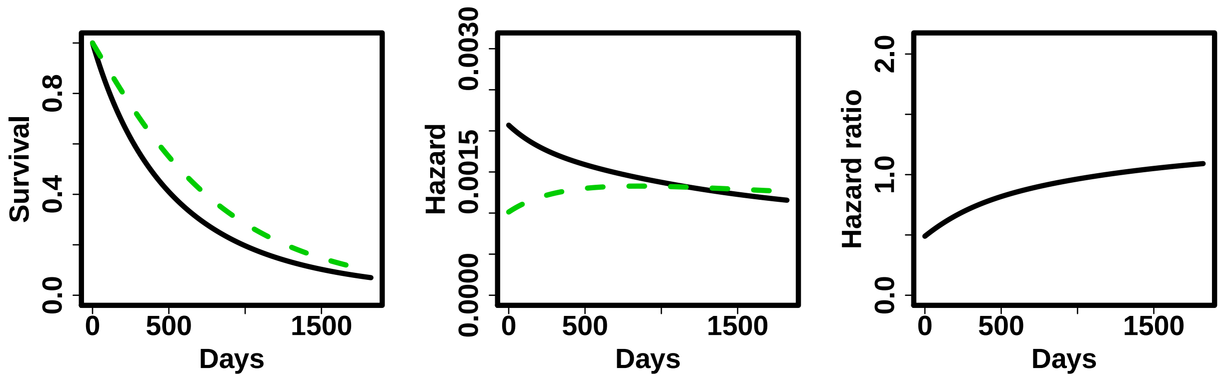
Survival
0.0
0.4
0.8
0
500
1500
Days
Hazard
0.0000
0.0015
0.0030
0
500
1500
Days
Hazard ratio
0.0
1.0
2.0
0
500
1500
Days
